# Transcriptomic Analysis for Differentially Expressed Genes in Ovarian Follicle Activation in the Zebrafish

**DOI:** 10.3389/fendo.2018.00593

**Published:** 2018-10-11

**Authors:** Bo Zhu, Lakhansing Pardeshi, Yingying Chen, Wei Ge

**Affiliations:** ^1^Centre of Reproduction, Development and Aging, Faculty of Health Sciences, University of Macau, Taipa, China; ^2^Genomics and Bioinformatics Core, Faculty of Health Sciences, University of Macau, Taipa, China

**Keywords:** transcriptomics, RNA-seq, follicles, folliculogenesis, puberty, zebrafish

## Abstract

In teleosts, the onset of puberty in females is marked by the appearance of the first wave of pre-vitellogenic (PV) follicles from the pool of primary growth (PG) follicles (follicle activation) in the ovary during sexual maturation. To understand the mechanisms underlying follicle activation and therefore puberty onset, we undertook this transcriptomic study to investigate gene expression profiles in the event. Our analysis revealed a total of 2,027 up-regulated and 859 down-regulated genes during the PG-PV transition. Gene Ontology (GO) analysis showed that in addition to basic cellular functions such as gene transcription, cell differentiation, and cell migration, other biological processes such as steroidogenesis, cell signaling and angiogenesis were also enriched in up-regulated genes; by comparison, some processes were down-regulated including piRNA metabolism, gene silencing and proteolysis. Further Kyoto Encyclopedia of Genes and Genomes (KEGG) analysis identified a variety of signaling pathways that might play pivotal roles in PG-PV transition, including MAPK, TGF-β, Hedgehog, FoxO, VEGF, Jak-STAT, and phosphatidylinositol signaling pathways. Other pathways of particular interest included endocytosis and glycosaminoglycan biosynthesis. We also analyzed expression changes of genes expressed in different compartments viz. oocytes and follicle cells. Interestingly, most oocyte-specific genes remained unchanged in expression during follicle activation whereas a great number of genes specifically expressed in the follicle cells showed significant changes in expression. Overall, this study reported a comprehensive analysis for genes, biological processes and pathways involved in follicle activation, which also marks female puberty onset in the zebrafish when occurring for the first time in sexual maturation.

## Introduction

In each mammalian ovarian cycle, a limited number of primordial follicles are recruited to undergo a gonadotropin-independent growth phase (primary and secondary follicles) leading to antral formation (initial recruitment). This is followed by gonadotropin-dependent selection toward formation of pre-ovulatory follicles (cyclic recruitment) (1). As a critical stage of ovarian folliculogenesis, follicle recruitment or activation has been extensively studied in different mammalian models. Despite this, our understanding of the event and its controlling mechanisms is still limited (2), partly due to the limited supply of early follicles for study.

Similar to that in mammals, the process of folliculogenesis in fish has also been divided into gonadotropin-independent stage (primary growth, PG) and gonadotropin-dependent stage (secondary growth, SG), which are equivalent to preantral and antral stages in mammals respectively (3), although this view has recently been revised by emerging genetic data (4). During fish life cycle, the first transition from PG to SG in the ovary is considered an early visible marker for puberty onset or activation of the hypothalamic-pituitary-gonad (HPG) axis in females (5–7). Although the PG-SG transition has been described in many fish species, the mechanisms that control this important developmental event remain poorly understood. Studies in different fish species have provided lines of evidence for roles of pituitary hormones especially gonadotropins (FSH and LH), gonadal steroids and growth factors in regulating the event. A classical experiment of hypophysectomy in the goldfish showed an arrest of folliculogenesis at the transition from PG to previtellogenic (PV, the beginning of SG with cortical alveoli) stage without pituitary, strongly suggesting important roles for pituitary hormones in controlling follicle activation (8). This idea has been supported by studies in other species. For example, PG follicle growth and PG-PV transition in salmons were associated with increased plasma FSH level and gonadal FSH receptor expression (9, 10). Similar results have also been reported in the zebrafish (11, 12). Our recent study in the zebrafish using gene knockout approach provided further genetic evidence for the importance of gonadotropin signaling, especially FSH-FSHR pathway in controlling follicle activation (4, 13). In addition to pituitary gonadotropins, gonadal steroids especially androgens have also been implicated in regulating early follicle growth and possibly activation as well. In the shortfinned eel (*Anguilla australis*), treatment with fish-specific androgen 11-ketotestosterone (11-KT) significantly promoted the growth of PG follicles *in vitro* (14) and PG-PV transition *in vivo* (15). Similar results have also been reported in the Atlantic cod (*Gadus morhua* L.) (16, 17). Recent *in vitro* studies in the coho salmon (*Oncorhynchus kisutch*) confirmed the stimulatory effect of 11-KT on PG follicles and their transition to PV follicles. Interestingly, these studies provided evidence for sequential roles of androgens and estrogens in early follicle development. Androgens [11-KT and testosterone (T)] but not estrogens (E2) promoted the growth of PG follicles and their transition to PV stage; however, E2 exhibited strong stimulatory effect on the development of PV follicles after the transition (18, 19). Evidence has also accumulated for roles of growth factors in controlling early folliculogenesis. Both IGF-I and II increased their plasma levels during PG-PV transition together with FSH and E2 (10). In agreement with this, IGF-I, but not insulin and leptin, increased the size of eel PG follicles *in vitro* (14).

As a daily spawner with continual development of ovarian follicles, the zebrafish has become a popular model for studying follicle development and its controlling mechanism (20). In a recent study, we demonstrated that the female zebrafish entered puberty when the first cohort of PV follicles appeared in the ovary. This happened when the body weight reached 100 mg or standard body length above 1.8 cm, suggesting a role for the somatotrophic or growth axis in activating the reproductive axis (7), similar to the situation in salmons (10) and mammals (21). By using candidate gene approach, a variety of genes especially growth factors and their receptors have been shown to display differential expression patterns during the PG-PV transition in zebrafish, including activin-inhibin-follistatin family (22, 23), transforming growth factor β (TGF-β) family (24), bone morphogenetic protein (BMP) family (25–28), epidermal growth factor (EGF) family (29–31), insulin-like growth factor (IGF) family (32–34), Kit family (35), and anti-Müllerian hormone (AMH) (36). The candidate gene approach has recently been boosted by the emergence of powerful genome editing technologies such as TALEN and CRISPR/Cas9. An increasing number of genes have been analyzed for their functions in early folliculogenesis in fish models. For example, disruption of oocyte-specific transcription factor *figla* in zebrafish (37) and ovarian aromatase (*cyp19a1a*) in zebrafish (28, 38, 39), medaka (40) and tilapia (41) blocked early folliculogenesis and induced female-to-male sex reversal. In addition, inactivation of *foxl2a/b* in zebrafish (42) and tilapia (41), *sf1* in tilapia (43), *foxl3* in medaka (44), and *bmp15* in zebrafish (28) all resulted in female-to-male sex reversal. Interestingly, the loss of *fshr* in female zebrafish caused complete failure of follicle activation or PG-PV transition followed by sex reversal to males (4, 45).

While the candidate target gene approach is still the major form of study for gene functions in folliculogenesis, there has been an increasing number of studies using high-throughput approaches such as transcriptomics using either microarray or next generation sequencing (NGS)-based RNA sequencing (RNA-seq) to investigate differentially expressed genes (DEGs) and regulatory networks. In humans, thousands of genes were shown by microarray analysis to be differentially expressed between mature oocytes and the somatic tissues (46). Similar approach also revealed hundreds of DEGs between the cumulus granulosa cells and the granulosa cells in the follicular fluid (47). Similar transcriptome studies have also been performed in a variety of mammalian species on temporal expression profiles at different stages of folliculogenesis, including bovine follicles of different sizes (48), primary and secondary follicles in sheep (49), secondary and early antral (tertiary) follicles in the goat (50), three different stages of antral follicles (small, medium and large) in the pig (51), and unassembled, primordial and primary follicles in the rat (2). In contrast to mammalian models, similar studies are limited in non-mammalian species. In the pigeon, pre-ovulatory and post-ovulatory ovaries were analyzed for transcriptomes, and more than 400 DEGs were identified (52). In teleosts, a few studies on ovarian transcriptomes have been reported using subtractive hybridization, serial analysis of gene expression (SAGE), microarray, or RNA-seq. In the flatfish, microarray analysis on three stages of follicle development, i.e., oocyte growth (vitellogenesis), maturation and follicle atresia, identified 118 DEGs (53). In the rainbow trout, three stages of precocious ovaries were analyzed for DEGs as compared to normal ovary by subtractive hybridization and microarray (54). A recent RNA-seq study in the common carp examined gene expression profiles at three stages of ovarian development, namely undifferentiated gonads, immature juvenile ovary and mature adult ovary, with a large number of DEGs assigned to biological processes related to reproduction (55). In the zebrafish, an early study used SAGE to analyze the transcriptome of full-grown follicles and compared it with similar transcriptomes of other vertebrate species (56). Despite these studies, there is a lack of transcriptome analysis on early folliculogenesis, which is critical for understanding the underlying mechanisms controlling follicle activation and therefore puberty onset. Using subtractive hybridization, a study in the coho salmon compared PG (perinucleolar stage) and PV follicles (early stage of SG with cortical alveoli) with the aim to identify DEGs between the two stages in early folliculogenesis. Some differentially expressed genes were identified, including zona pellucida glycoprotein (*zp*) genes, vitellogenin receptors (*vldlr*), anti-Müllerian hormone (*amh*), gonadal soma-derived growth factor (*gsdf* ), and follicle-stimulating hormone receptor (*fshr*) (57). Also in the coho salmon, a recent study demonstrated significant changes in ovarian transcriptome in response to 11-KT, which stimulates PG-PV transition. The DEGs identified included those involved in gonadotropin, steroid and growth factor signaling (19).

Using zebrafish as the model, we undertook this transcriptomic study to analyze gene expression profiles during follicle activation/recruitment or PG-PV transition by NGS-based RNA-seq. This high-throughput approach of gene expression analysis promises to provide us a comprehensive and global view on genes and regulatory networks or pathways involved in follicle recruitment or activation. The data obtained from the present study together with the availability of genome editing technologies in the zebrafish will make this species an excellent platform for studying gene functions in ovarian folliculogenesis.

## Materials and methods

### Animals

The wild type zebrafish (*Danio rerio*) of AB strain was used in this study. The fish were maintained in the ZebTEC multilinking rack system (Tecniplast; Buguggiate, Italy) under an artificial photoperiod of 14 h light:10 h dark. The temperature, pH and conductivity of the system were 28 ± 1C°, 7.5 and 400 μS/cm, respectively. The fish were fed with paramecia, brine shrimp, and Otohime fish diet (Marubeni Nisshin Feed, Tokyo, Japan) twice a day during larval, juvenile and adult stages, respectively.

All experiments were performed under a license from the Government of the Macau Special Administrative Region (SAR), and approved by the Animal Experimentation Ethics Committee of the University of Macau.

### Histological examination

For histological examination, the dissected ovarian samples were immediately fixed in Bouin's solution overnight at room temperature. The fixed samples were dehydrated and embedded in paraffin according to our previous report (6), and then serially cut into 7-μm sections on a microtome (Leica, Wetzlar, Germany). Slides were stained with hematoxylin and eosin (H&E), followed by observation and photographing on Nikon ECLIPSE Ni-U microscope (Nikon, Tokyo, Japan).

### Follicle isolation and RNA preparation

Zebrafish were anesthetized by ice shock and decapitated before dissection. Before follicle isolation, ovaries were carefully removed from three female zebrafish and placed in a 100-mm Petri dish containing 60% Leibovitz L-15 medium (Gibco Invitrogen, Carlsbad, CA). Fat tissue and ligaments surrounding the ovaries were stripped off using a BD Microlance 26G needle (BD, San Diego, CA). To separate follicles, a transfer pipette (JET Biofil, Guangzhou, China) was used to pipet ovarian fragments up and down a few times, followed by further pipetting with a BD 23G needle for a few times. After separation of follicles, the PG (<100 μm) and PV follicles (~180–250 μm) were isolated by filtering through sieves with different pore sizes, including 100 μm (SPL Lifesciences, Waunakee, WI), 180 and 250 μm (Jiu Feng, Heng Shui, China). The PG follicles that passed through the 100 μm sieve were collected by centrifugation at 3,000 rpm for 2 min, and the PV follicles retained between 180 and 250 μm were collected also by centrifugation. The PG and PV follicles were washed twice with 1 X PBS. In total, nine zebrafish were used and divided into three groups (3 fish per group) for follicle isolation and RNA preparation (*n* = 3). The entire process of ovary dissection, follicle isolation and washing was finished within 1–1.5 h to minimize changes of gene expression *in vitro*. Total RNA from PG and PV follicles of each group was extracted using Tri-Reagent (Molecular Research Center, Cincinnati, OH) according to the protocol of the manufacturer and our previous report (58). The RNA was then treated with DNase for 10 min at 37°C to remove genomic DNA [10 μg RNA in 100 μl reaction buffer with 2U DNase I from NEB (Ipswich, MA)] followed by phenol-chloroform extraction and ethanol precipitation.

### RNA sequencing and differential expression analysis

For RNA library construction, the integrity of the RNA samples was first analyzed on the Bioanalyser 2100 (Agilent, Stockport, UK). RNA libraries were prepared using the NEBNext Ultra Directional RNA Library Prep Kit (NEB) and sequenced on the HiSeq 2500 Sequencing System (Illumina, San Diego, CA) with 100-bp paired end reads. FastQC (v0.11.5) (https://www.bioinformatics.babraham.ac.uk/projects/fastqc/) was used for quality checking of the raw fastq data. These raw reads were aligned against zebrafish reference genome (assembly GRCz10) using TopHat2 (v2.1.1) (https://ccb.jhu.edu/software/tophat/manual.shtml) (59) with default parameters. StringTie (v1.3.3b) (https://ccb.jhu.edu/software/stringtie/index.shtml) (60, 61) and prepDE.py Python script provided with StringTie tool were used to assemble the alignments into transcripts and extract the raw read counts for reference genomic features respectively. The read count matrix was processed by DeSeq2 package (v1.16.1) (https://bioconductor.org/packages/release/bioc/html/DESeq2.html) (62) for differential gene expression analysis. Genes with *p*-values <= 0.05 and log2 (fold change) >= 1 were considered as differentially expressed genes (DEGs) with statistical significance.

The transcriptome data analysis was performed at the Genomics and Bioinformatics Core (Faculty of Health Sciences, University of Macau), and all the raw data were submitted to NCBI GEO database with accession number GSE99308.

### Functional enrichment analysis

Significant DEGs were subject to GO and KEGG pathway enrichment analysis using online resource DAVID (v6.8) (63). GO terms and KEGG pathways with *p*-values < 0.05 were considered significantly enriched. DEGs with significant Log2 (fold change) were mapped onto the enriched KEGG pathway maps using Pathview package (v1.16.5) (http://bioconductor.org/packages/release/bioc/html/pathview.html) (64).

### Reverse transcription and real-time quantitative PCR (RT-qPCR)

Reverse transcription was performed at 37°C for 2 h in a total volume of 10 μl reaction solution containing 3 μg RNA, 0.5 μg oligo (dT), 1X MMLV RT buffer, 0.5 mM each dNTP, 0.1 mM dithiothreitol, and 100 U M-MLV reverse transcriptase (Invitrogen, Carlsbad, CA). To validate the RNA-seq data, we determined the expression levels of some selected genes by RT-qPCR in both PG and PV follicles, including *fshr, cyp19a1a, inha, inhbaa, notch3, amh, gadd45ga, lpl, march4, ube2ql1, zp2.1*, and *zp2.5*. The expression levels were normalized to that of the housekeeping gene *ef1a*. The standard for each gene was prepared by PCR amplification of cDNA fragments with specific primers (Supplemental Table 1). After purification from the gel, the amplicons were quantitated by electrophoresis along with O'GeneRuler DNA Ladder Mix (Thermo Scientific, Waltham, MA), and the copy number of DNA molecule was calculated. The real-time qPCR assay was performed on the CFX96 Real-time PCR Detection System (Bio-Rad, Hercules, CA) and repeated twice.

### Data analysis

The mRNA level of each target gene was normalized to the internal control *ef1a*. All values were expressed as the mean ± SEM (*n* = 3), and the data were analyzed by *t*-test using Prism 6 on Macintosh OS X (GraphPad Software, San Diego, CA).

## Results

### Differential gene expression profiles in PG and PV follicles

**Figure 1:**
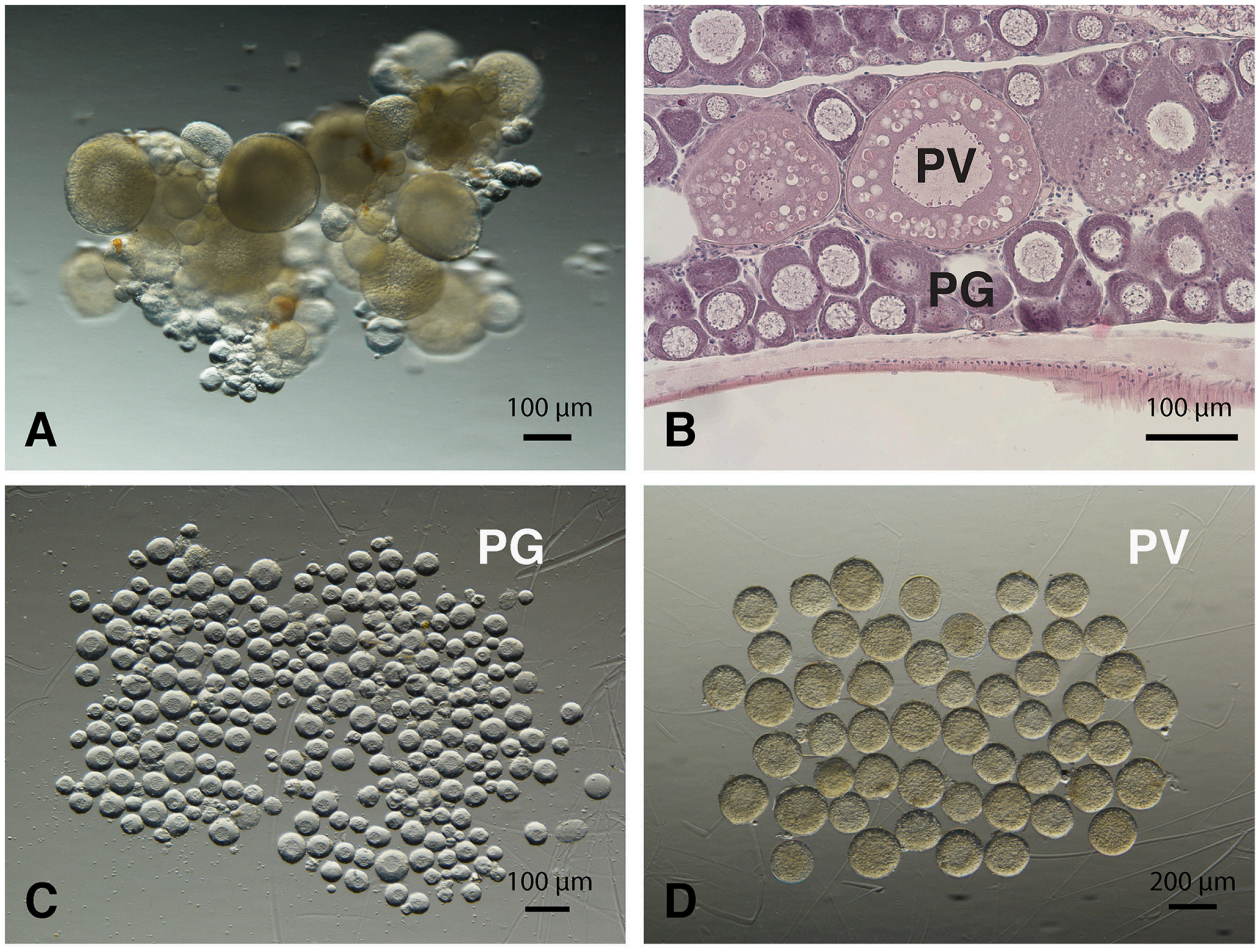
Morphology of zebrafish PG and PV follicles. **(A)** Ovarian fragments with PG and PV follicles. **(B)** Histological morphology of PG and PV follicles. **(C)** Isolated PG follicles (< 100 μm in diameter). **(D)** Isolated PV follicles (180 to 250 μm in diameter).

**Figure 2:**
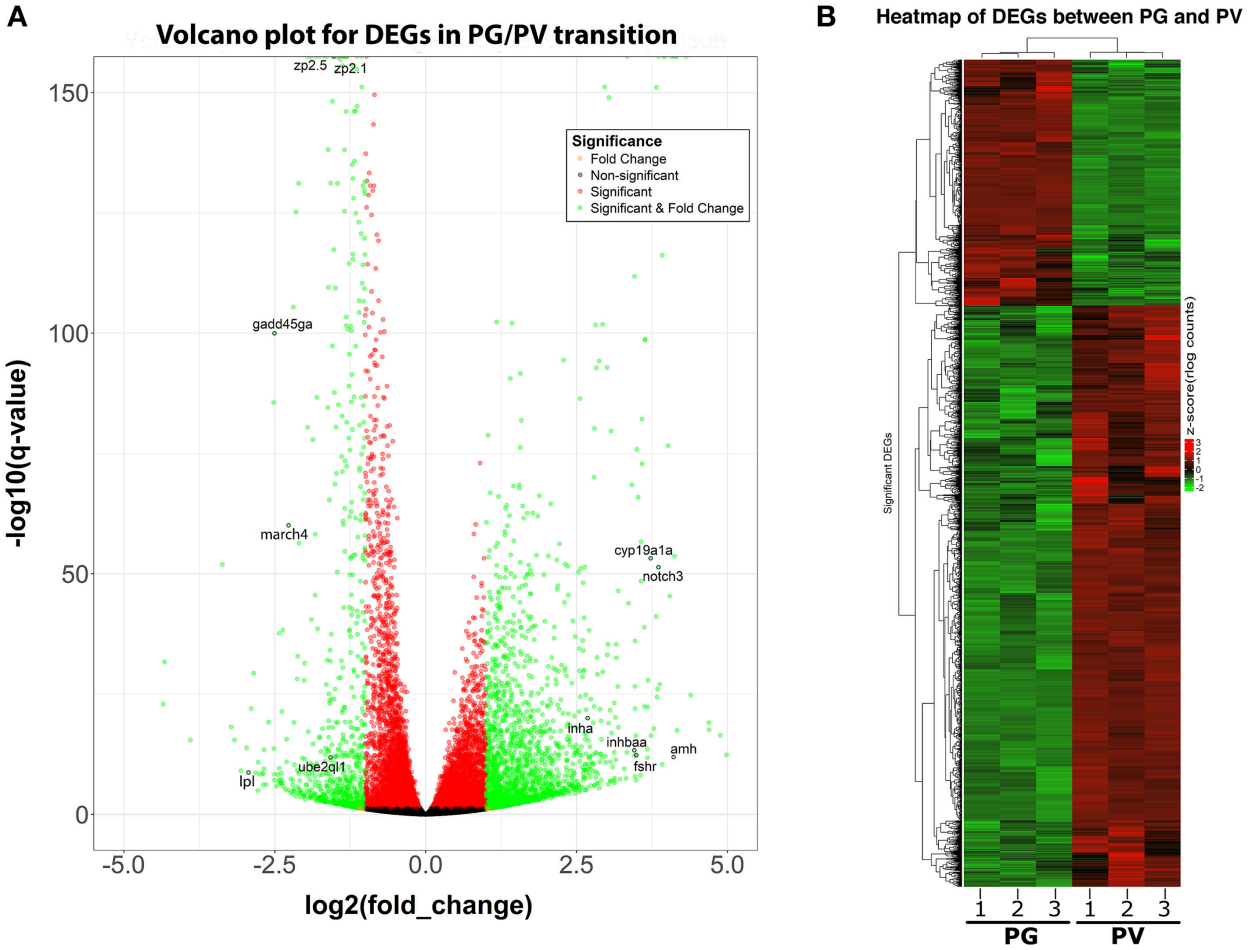
Volcano plot and heatmap for DEGs between PG and PV follicles. **(A)** Volcano plot for DGEs in PG/PV transition, showing distribution of significance [-log10(*q*-value)] vs. fold change [log2(fold change)] for all genes. The genes selected for qPCR validation are highlighted with black circles and gene symbols marked, including 6 down-regulated and 6 up-regulated genes. Genes in green are significant DEGs [*q*-value < = 0.05 and log2(fold change) > = 1]. **(B)** Heatmap of significant DGEs between PG and PV stage. Regularized log transformed (rlog) count matrix was generated using DeSeq2 package. Significant DEGs were used to plot the heatmap of rlog counts.

**Figure 3:**
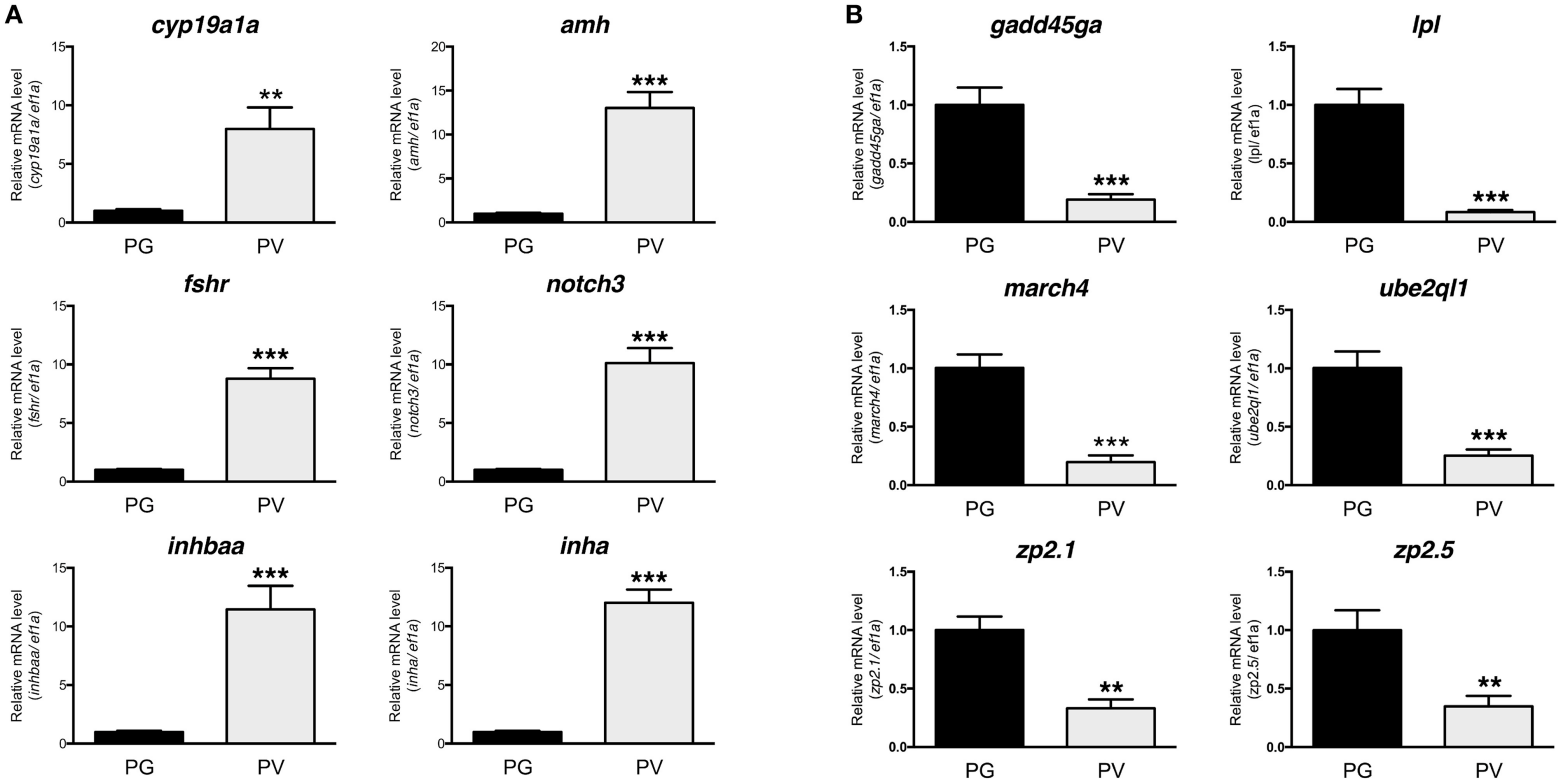
Validation of RNA-seq data by real-time qPCR on 12 significant DEGs highlighted in the Volcano plot, including 6 up-regulated and 6 down-regulated DEGs. **(A)** Up-regulated DGEs (PG vs. PV): *cyp19a1a, amh, fshr, notch3, inhbaa*, and *inha*. **(B)** Down-regulated DGEs (PG vs. PV): *gadd45ga, lpl, march4, ube2ql1, zp2.1*, and *zp2.5*. All the selected DGEs showed the same expression pattern in both RNA-seq and qPCR analyses. **P < 0.01; ***P < 0.001.

The PG and PV follicles were isolated as described. The PG follicles were small and transparent with diameters less than 100 μm, whereas the PV follicles were much larger and less transparent. The PV follicles collected for analysis ranged from 180 to 250 μm in diameter. In histological sections, the PV follicles were bigger with numerous cortical alveoli in the oocytes and a continual layer of follicle cells surrounding the oocyte (Figure 1). Sequence analysis of mRNAs from PG and PV follicles showed that on average 84% reads were aligned to the zebrafish reference genome. The DEGs between PG and PV follicles are shown in four groups in the Volcano plot based on log2(fold change) and *q*-value (Benjamini-Hochberg adjusted *p*-value) [-log10(*q*-value)]: (1) significant for both fold change and *q*-value, (2) significant for fold change but nonsignificant for *q*-value, (3) nonsignificant for fold change but significant for *q*-value, and (4) nonsignificant for both fold change and *q*-value (Figure 2A). A large number of genes showed significant differential expression between the two stages (Figure 2B). To confirm the RNA-seq result, we evaluated 6 up-regulated genes (*cyp19a1a, fshr, inha, inhbaa, notch3*, and *amh*) and 6 down-regulated genes (*gadd45ga, lpl, march4, ube2ql1, zp2.1*, and *zp2.5*) (marked in Figure 2A) using RT-qPCR. The result showed that all these genes exhibited significant difference in their expression between PG and PV stages, fully agreeing with the RNA-seq result (Figure 3).

**Figure 4:**
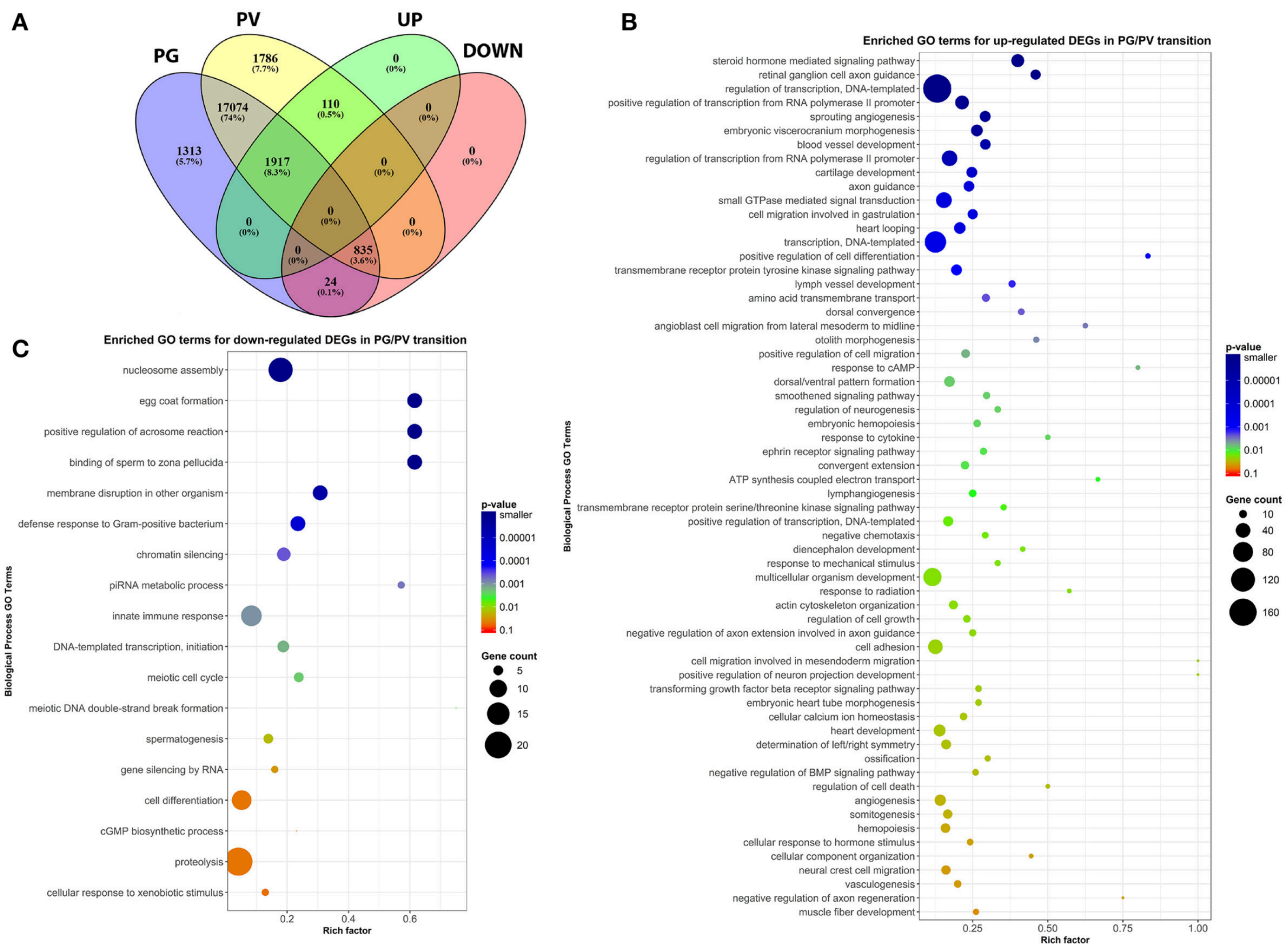
Venn diagram and scatter plots of enriched GO terms for up-regulated and down-regulated DEGs in PG/PV transition. **(A)** Venn diagram showing distribution of significant DEGs in different classes, including groups for expressed genes in PG and PV follicles, and up- and down-regulated gene groups. Among the genes expressed in both PG and PV follicles, 17,074 genes showed no significant difference in expression, and 1,917 and 835 were significantly up- and down-regulated, respectively, from PG to PV stage. A total of 1,786 genes were detected in PV follicles only, and 110 were significantly up-regulated in PG/PV transition. On the other hand, a total of 1,313 genes were detected in PG follicles only, and 24 were significantly down-regulated genes in PG/PV transition. **(B)** Scatter plot for enriched GO terms for up-regulated DEGs in PG/PV transition. **(C)** Scatter plot for enriched GO terms for down-regulated DEGs in PG/PV transition. Rich factor is the ratio of number of DEGs for particular GO term over the number of genes in background for that GO term. The size and color of the circles in scatter plots are scaled with respect to the number of DEGs and *p*-value respectively.

To classify the genes expressed in PG and PV follicles, including total expressed genes and DEGs (both up- and down-regulated genes), we generated the Venn diagram by an online software (http://bioinformatics.psb.ugent.be/webtools/Venn/). In total, 20,173 genes were detected with 17,074 expressed in both PG and PV follicles, and 1,313 and 1,786 detected in PG and PV only respectively. Among the genes expressed in both stages, 1,917 and 835 genes were up- and down-regulated in PV follicles respectively with statistical significance in both fold change and *q*-value. For 1,786 genes specifically expressed at PV stage, 110 showed significance in in both fold change and *q*-value whereas 24 PG-specific down-regulated genes showed significance (Figure 4A; for details see Supplemental Table 2). All these DEGs with significant change were subject to further GO analysis.

### GO analysis for DEGs in PG and PV follicles

After analysis of gene expression profiles in PG and PV follicles, the DEGs with statistical significance were subject to further GO enrichment analysis, which covered all 2,027 (1917 + 110) up-regulated and 859 (835 + 24) down-regulated genes mentioned above. Among the three GO domains, we focused our analysis on the domain of Biological Process (BP).

**Figure 5:**
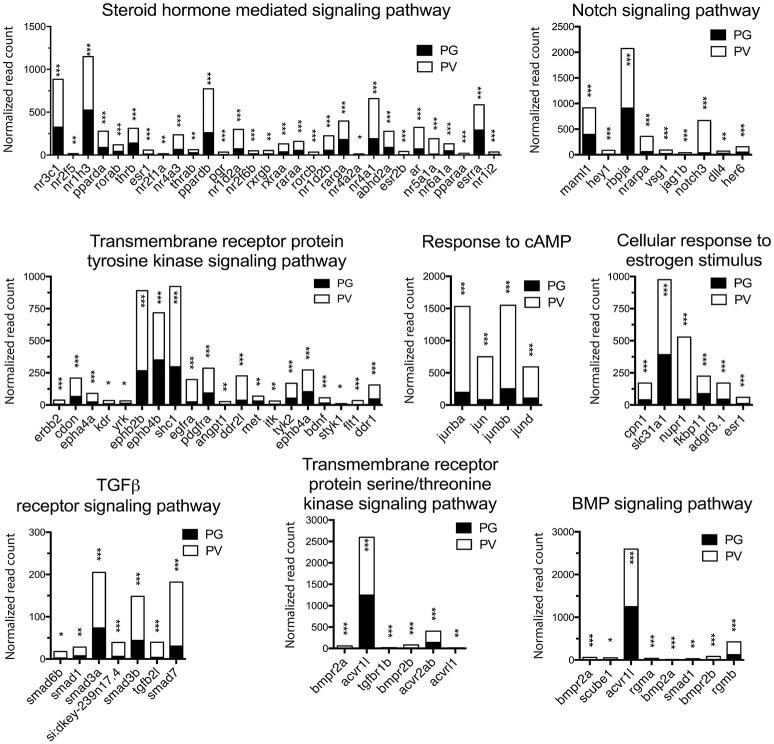
Graphic illustration of expression levels of various DEGs up-regulated in different biological processes related to cell signaling in PV follicles. The GO terms chosen for plotting were those with *P* < 0.1. *P < 0.05; **P < 0.01; ***P < 0.001.

**Figure 6:**
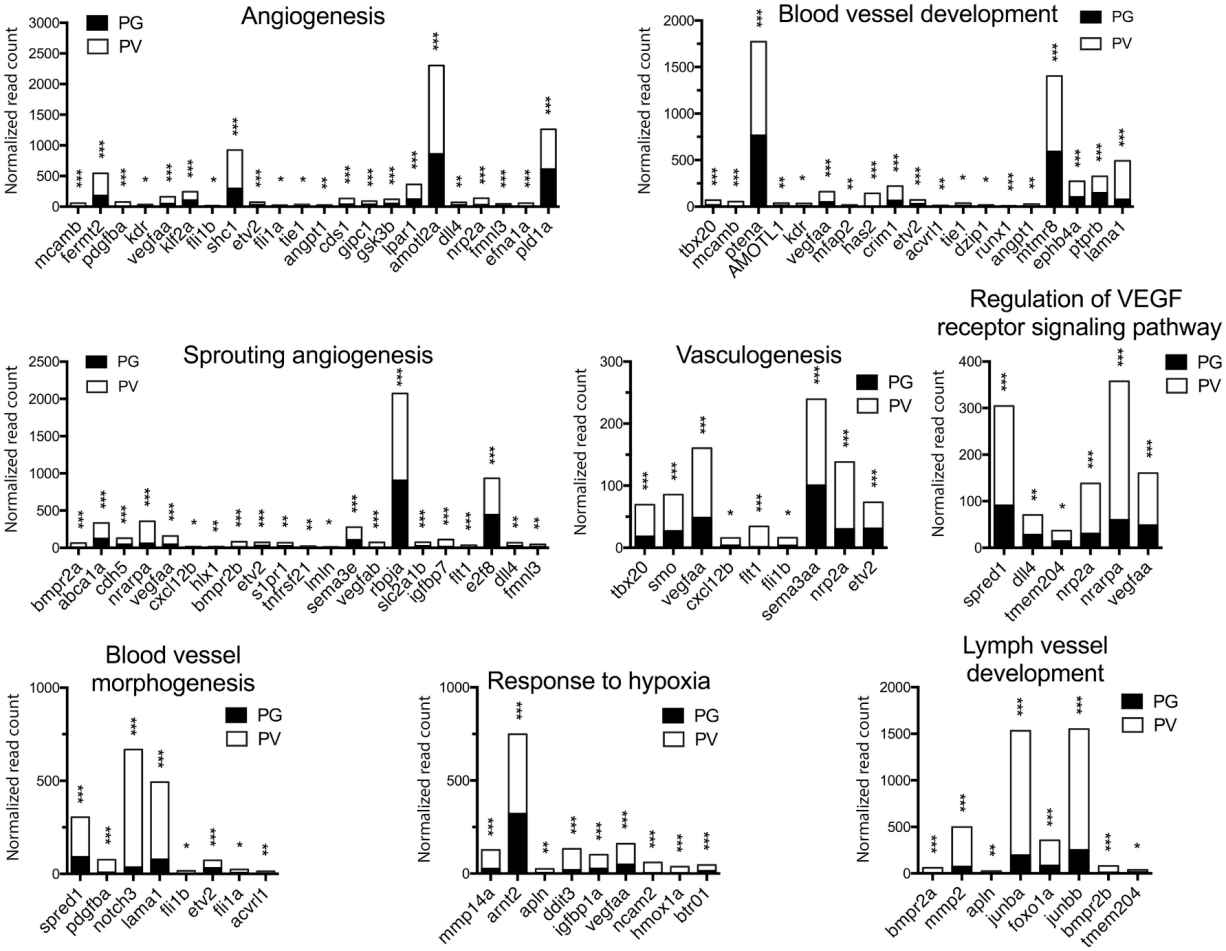
Graphic illustration of expression levels of various DEGs up-regulated in different biological processes related to angiogenesis and blood vessel formation in PV follicles. The GO terms chosen for plotting were those with *P* < 0.1. *P < 0.05; **P < 0.01; ***P < 0.001.

**Figure 7:**
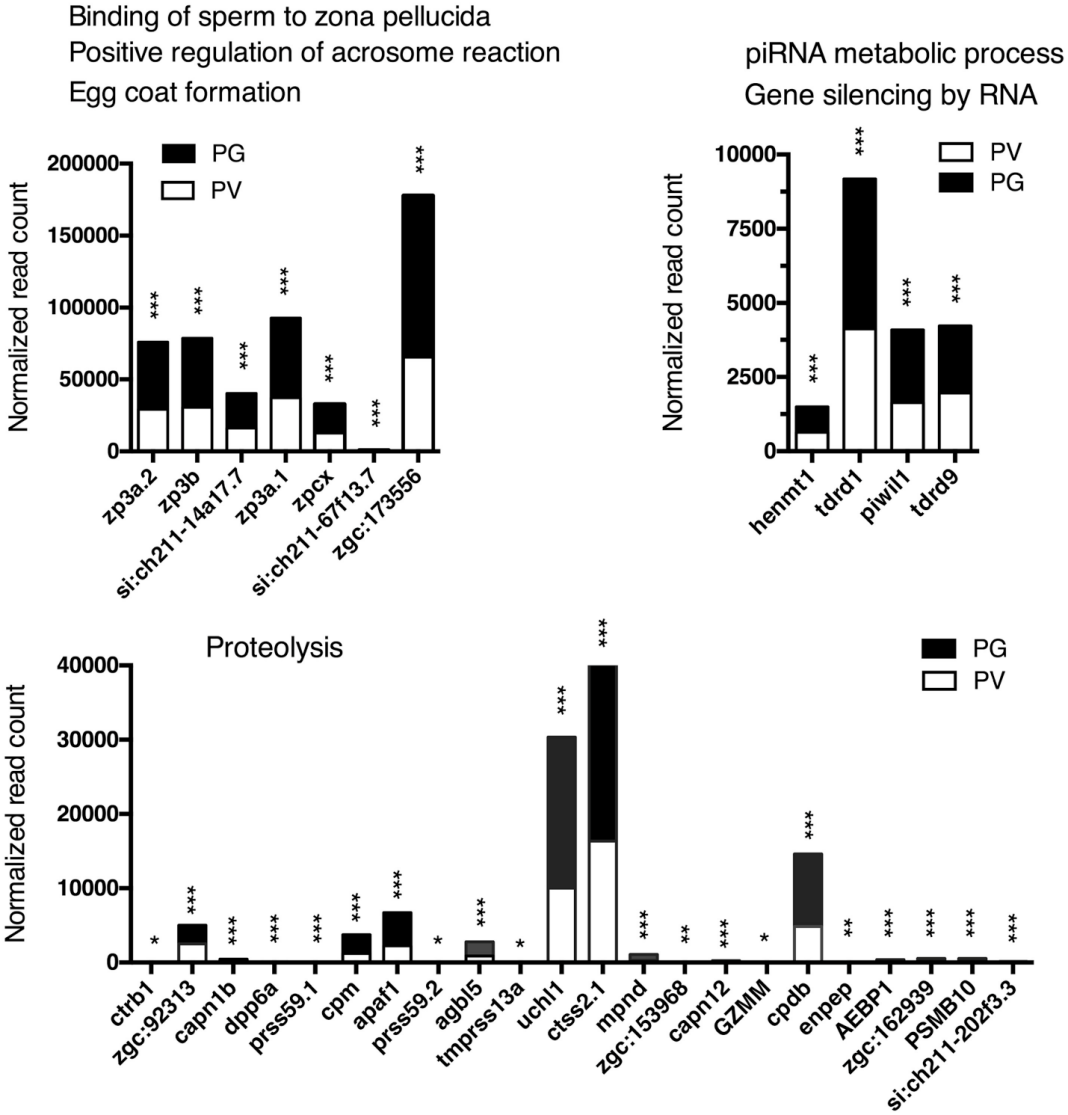
Graphic illustration of expression levels of various DEGs down-regulated in different biological processes related to egg coat formation and egg-sperm interaction, piRNA metabolism and RNA silencing, and proteolysis in PV follicles. The GO terms chosen for plotting were those with *P* < 0.1. *P < 0.05; **P < 0.01; ***P < 0.001.

The analysis revealed significant enrichment of a large number of GO terms during PG-PV transition. As expected, many GO terms associated with fundamental biological processes were enriched for up-regulated genes during follicle activation, including transcription (e.g., *DNA-templated transcription*), translation (e.g., *amino acid transmembrane transport*), metabolism (e.g., *ATP synthesis coupled electron transport*), and key cellular activities (*cell growth, differentiation, migration, adhesion*, and *death*). In addition, a variety of key signal transduction processes were enhanced, as indicated by the enrichment of up-regulated genes in GO terms associated with *steroid hormone mediated signaling pathway, small GTPase mediated signal transduction, receptor tyrosine kinase signaling pathway, receptor serine/threonine kinase signaling pathway, TGF-*β *receptor signaling pathway, BMP signaling pathway, response to cAMP, response to cytokine, smoothened signaling pathway, ephrine receptor signaling pathway*, and *cellular response to hormone stimulus* (Figure 4B). The up-regulated genes of some of these GO terms and their expression levels are illustrated in Figure 5. Interestingly, a series of GO terms associated with angiogenesis and vascularization were also up-regulated during follicle activation, including *angiogenesis, sprouting angiogenesis, lymphangiogenesis, lymph vessel development, vasculogenesis*, and *blood vessel development* (Figures 4B, 6). While a large number of GO terms were up-regulated during PG-PV transition, a number of GO terms were enriched for down-regulated genes, and they are mostly associated with chromatin structure (e.g., *nucleosome assembly* and *chromatin silencing*), egg-sperm interaction (e.g., *egg coat formation, acrosome reaction*, and *sperm binding*), RNA silencing (e.g., *piRNA metabolic process* and *gene silencing by RNA*), meiosis (e.g., *meiotic cell cycle* and *meiotic DNA double-strand break formation*), and *proteolysis* (Figure 4C). The representative GO terms enriched for down-regulated genes and the expression levels of these genes are plotted in Figure 7.

### Pathway analysis for DEGs in PG and PV follicles

**Figure 8:**
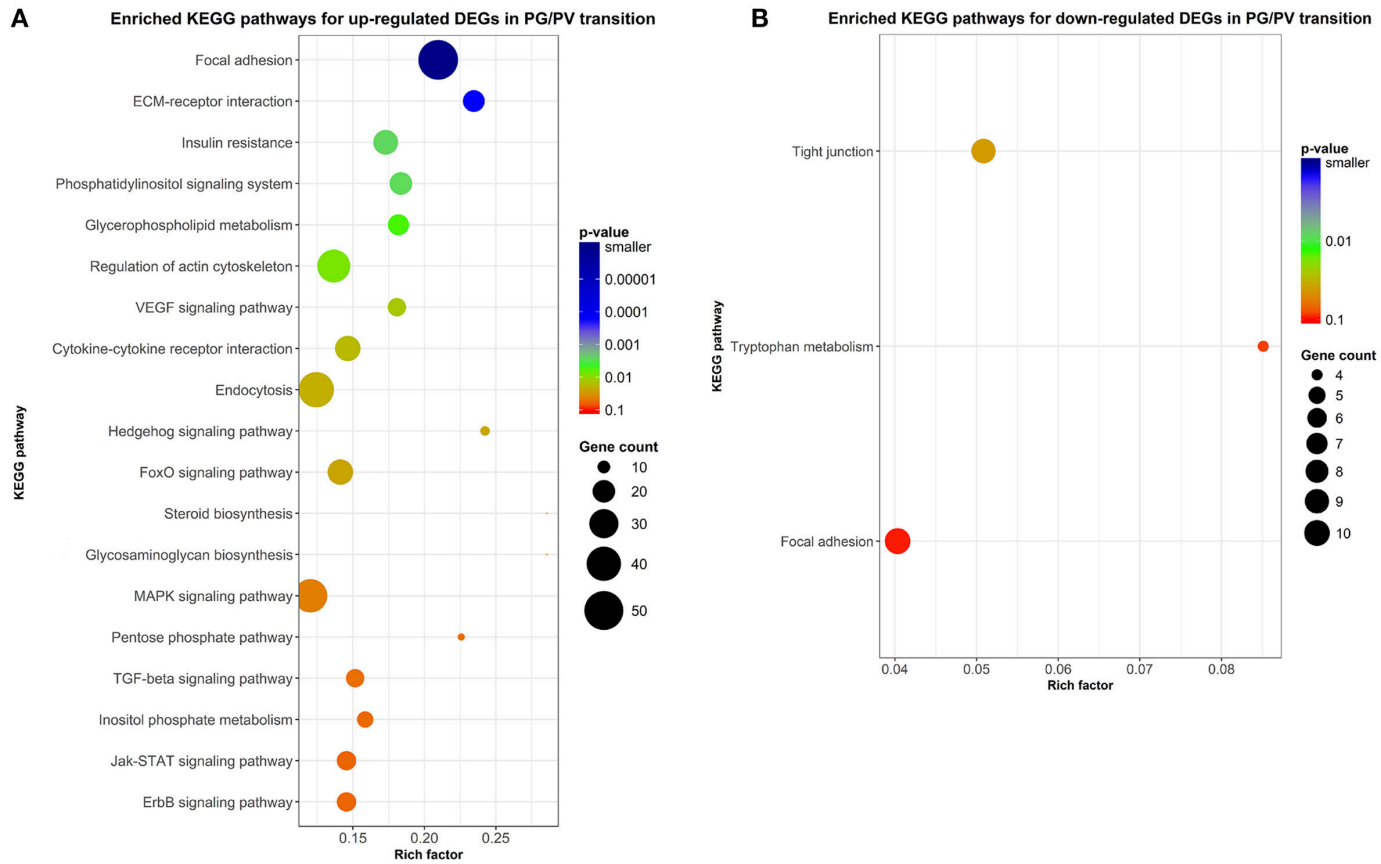
Scatter plots of enriched KEGG pathways. **(A)** Enriched KEGG pathways for up-regulated DEGs in PG/PV transition. **(B)** Enriched KEGG pathways for down-regulated DEGs in PG/PV transition. Rich factor is the ratio of number of DEGs for particular KEGG pathway over the number of genes in background for that KEGG pathway. The size and color of the circles in the plots are scaled with respect to the number of DEGs and *p*-value respectively.

In addition to enrichment analysis for GO terms, we also performed KEGG pathway analysis to identify pathways potentially involved in follicle activation. Nineteen pathways were found to be significantly enriched for genes up-regulated during PG-PV transition (*p* < 0.05). Interestingly, most of these pathways are involved in signal transduction, including phosphatidylinositol signaling pathway, VEGF signaling pathway, hedgehog signaling pathway, FoxO signaling pathway, MAPK signaling pathway, TGF-β signaling pathway, Jak-STAT signaling pathway, ErbB signaling pathway, ECM-receptor interaction and cytokine-cytokine receptor interaction (Figure 8A). Several metabolic pathways were also enriched for up-regulated DEGs, including glycerophospholipid metabolism and glycosaminoglycan biosynthesis. Interestingly, a large number of up-regulated genes belonged to the pathway of endocytosis (Figure 8A). Compared with up-regulated pathways, fewer pathways were found to be down-regulated including tight junction and tryptophan metabolism (Figure 8B). The enriched KEGG pathways for both up- and down-regulated genes are shown in the Supplemental Figures 1, 2, respectively.

### Expression of oocyte- and follicle cell-specific genes during follicle activation

Ovarian follicles consist of oocytes and somatic follicle cells. According to our published and unpublished data, the oocyte-specific genes often showed little changes in expression during follicle activation, whereas those expressed in the follicle cells are often up- or down-regulated. To provide further evidence for this observation, we examined the expression changes of genes specifically expressed in the oocytes and follicle cells. Since this issue has not been systematically studied in the zebrafish, the information about the localization of gene expression in the two compartments was based on previous studies in the zebrafish and a dataset available in the sheep (65), which was the first comprehensive RNA-seq study in mammals on transcriptomes of oocyte and granulosa cells in early follicles.

**Figure 9:**
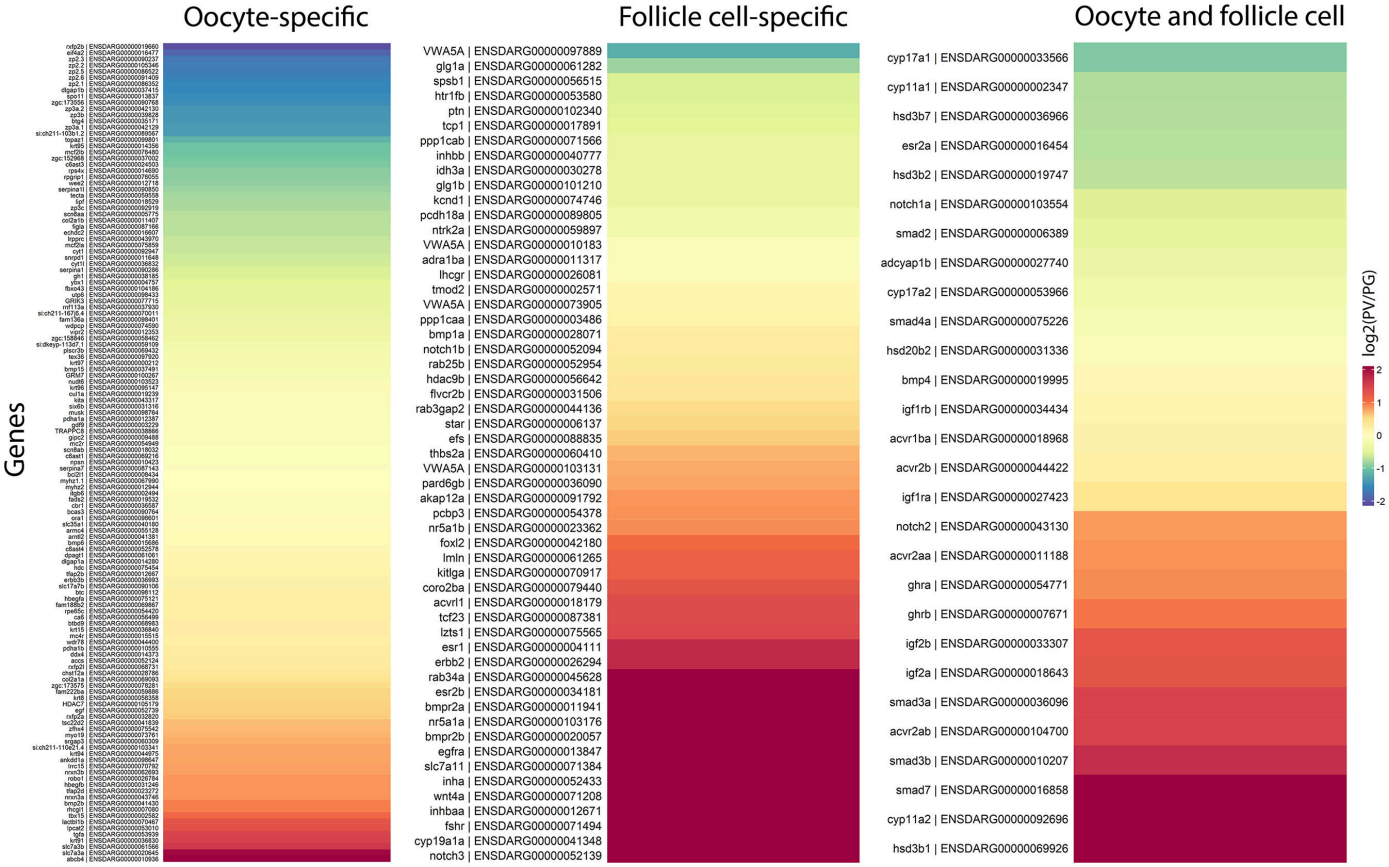
Heatmaps of gene expression change during PG/PV transition with respect to cell type specificity in the ovarian follicle, viz. oocytes and follicle cells. The genes in red indicates those that increased expression from PG to PV whereas those in blue decreased expression in the process.

In agreement with our observations, most of genes expressed in the oocytes showed stable expression from PG to PV stage, including some well-known oocyte factors such as *kita* (Kit receptor A) and *ybx1* (Y box binding protein 1). Some oocyte-specific genes showed decreased expression, including *rxfp2b* (Insl3 receptor)*, spo11* (initiator of meiotic double stranded breaks), and members of zona pellucida (ZP) family proteins, whereas some were increased such as *abcb4* (a member of the ATP-binding cassette transporter family) and *slc7a3a/b* (cationic amino acid transporter 3). In contrast, a large number of genes expressed in the follicle cells displayed significant changes in their expression during the PG-PV transition and most were up-regulated. Many of the top up-regulated genes are related to steroidogenesis, synthesis of estrogens and their actions, and signaling of various endocrine and paracrine factors, including *nr5a1a/b* (steroidogenic factor 1), *cyp19a1a* (ovarian aromatase) and its transcription stimulator *foxl2* (forkhead box L2), *esr1* and *esr2b* (estrogen receptors), *fshr* (FSH receptor), *notch3* (neurogenic locus notch homolog protein 3)*, inha* and *inhbaa* (inhibin and activin subunits), *acvrl1* (a type I receptor of TGF-β superfamily), *wnt4a* (a ligand of frizzled family receptors), *egfra* and *erbb2* (receptors of EGF family), *bmpr2a/b* (type II receptors of BMPs), and *kitlga* (Kit ligand A) (Figure 9).

## Discussion

The follicle development in the zebrafish consists of two major phases, i.e., PG and SG. The SG phase is further divided into previtellogenic (PV), early vitellogenic (EV), mid-vitellogenic (MV), late vitellogenic (LV), and full-grown (FG) stages according to follicle size and morphological characteristics (3, 66, 67). Although zebrafish is now being commonly used as a model for studying fish reproduction and its folliculogenesis in the ovary is well characterized at morphological level, the molecular mechanisms underlying follicle activation or PG-PV transition remain largely unknown. Using NGS-based RNA-seq, the present study demonstrated, for the first time, a comprehensive gene expression profile during ovarian follicle activation in the zebrafish. Our data showed that although most of the genes detected were common between the two follicle stages, many genes showed stage specificity in expression. Our analysis revealed a large number of candidate genes that were potentially involved in the activation of ovarian follicles or PG-PV transition, which also marks puberty onset when it occurs for the first time in zebrafish life cycle.

The quality of RNA-seq analysis was validated by real-time qPCR on twelve selected genes with six up- and six down-regulated from PG to PV. These genes were chosen based on either their positions in the Volcano plot or previous studies. All twelve genes showed consistent results in RNA-seq and qPCR analyses. The up-regulation of *cyp19a1a, fshr, amh, inha* and *inhbaa* is consistent with previous studies (11, 23, 36, 68) and the increased expression of *notch3* agrees with our unpublished data (Lau and Ge, unpublished). A recent RNA-seq study in the coho salmon also demonstrated increased expression of *cyp19a1, fshr, amh* and *inha* in 11-KT-induced PG-PV transition (19). Interestingly, the expression of zona pellucida (Zp/*zp*) genes (*zp2* and *zp3*) showed significant reduction during PG-PV transition. This seems contradictory to the role of Zp proteins in follicle development because the chorion, which consists of Zp proteins, is one of the hallmarks for follicle activation or PG-PV transition (23). This pattern of *zp* expression, however, agrees well with previous studies in both zebrafish and other species. The transcripts of *zp2* and *zp3* were abundantly expressed in the PG oocytes, and their levels decreased significantly while entering the PV stage. This was also confirmed by *in situ* hybridization, transgenesis and promoter analysis (69–71). Furthermore, their expression was independent of estrogen signaling (70). This has been confirmed in other fish species such as coho salmon (57).

In addition to the aforementioned genes, further analysis of the RNA-seq data identified a large number of biological processes and pathways that are potentially involved in controlling follicle activation, including those concerning transcription, translation, metabolism, and various cellular activities such as proliferation, differentiation, migration, and death. These are expected because follicle activation involves significant structural remodeling. Herein we will focus our discussion on the following aspects: signal transduction, angiogenesis, endocytosis, glycosaminoglycan biosynthesis, RNA silencing, and proteolysis.

### Signal transduction

Follicle activation is a critical step of early folliculogenesis, which involves dramatic changes in both structure and function (1, 72). A large body of evidence has accumulated in recent years in both mammals and fish that follicle activation or recruitment involves extensive regulation by endocrine and paracrine factors (20, 73, 74), which activate a variety of signaling pathways (75). As master hormones that control gonadal development and function, the pituitary gonadotropins (FSH and LH) signal mostly through cAMP/PKA pathway (76, 77). Loss of FSH receptor (*fshr*) in the zebrafish led to complete arrest of folliculogenesis at PG stage (4), suggesting an important role for gonadotropin signaling at follicle activation. Our demonstration of increased *fshr* expression (67) and enrichment of biological process *response to cAMP* during PG-PV transition provides supportive evidence for such regulation.

Together with gonadotropins, steroid hormones are believed to be essential for normal folliculogenesis, in particular the estrogen signaling pathway (78, 79). The involvement of gonadal steroids in follicle activation is supported in the present study by enriched GO terms of steroid hormone mediated signaling pathway and cellular response to estrogen stimulus. Among the genes significantly up-regulated were nuclear estrogen receptors (*esr1* and *esr2b*), androgen receptor (*ar*) and progestin receptor (*pgr*). The increased expression of nuclear estrogen receptors agrees well with our recent study in the zebrafish (80) and the reports in the coho salmon that E2 had potent stimulatory effect on the growth of PV follicles and accumulation of cortical alveoli (18, 19). In mammals, androgen signaling has been reported to be essential for normal folliculogenesis (81), and the disruption of androgen signaling resulted in defective folliculogenesis (82). In fish, lines of evidence from different species also point to the importance of androgens in early follicle development, especially growth of PG follicles and PG-PV transition (14, 16–19). Using RNA-seq approach, a recent study in the coho salmon demonstrated significant changes in ovarian transcriptome after exposure to 11-KT. A variety of genes were up-regulated by 11-KT, including genes associated with steroidogenesis and steroid action (19), similar to our data in this study. In Japanese huchen and common carp, both progestin (DHP, 17α, 20β-dihydroxy-4-pregnen-3-one) and E2 have been shown to regulate early oogenesis (83). A recent study in the zebrafish showed that the disruption of *pgr* led to female infertility due to failed ovulation (84). It would be interesting to examine if the loss of *pgr* has any impact on early follicle development. Taken together, steroid-mediated signaling pathway might be one of the pivotal mechanisms that regulate PG-PV transition in the zebrafish ovary.

In addition to gonadotropins and steroids, a large number of local ovarian paracrine factors especially peptide growth factors have been reported to play roles in controlling mammalian folliculogenesis especially during early stages (76, 85), including families of IGF (86), EGF (87), TGF-β (88), activin (89), BMP including BMP15 and GDF9 (90), Kit (91), and Notch (92). These growth factors and their signaling components also exist in the zebrafish ovary (22, 24–27, 30, 34, 93, 94). The importance of these ovarian paracrine factors in controlling zebrafish follicle activation is strongly supported by our RNA-seq data. A series of GO terms concerning signal transduction pathways, mostly those in growth factor signaling, were enriched in up-regulated genes, including transmembrane receptor protein tyrosine kinase signaling pathway, TGF-β receptor signaling pathway, transmembrane receptor protein serine/threonine kinase signaling pathway, BMP signaling pathway, and Notch signaling pathway. Consistent with GO enrichment analysis, the KEGG pathway analysis also suggested seminal importance for various cellular signaling pathways in follicle activation. Most pathways with significant enrichment for up-regulated genes turned out to be signal transduction pathways, including phosphatidylinositol, VEGF, TGF-β, hedgehog, FoxO, MAPK, Jak-STAT, and ErbB signaling pathways. Most of these pathways have been implicated in ovarian follicle activation or recruitment in mammals (95–97). Among these pathways, the phosphatidylinositol or PI3K/PTEN/Akt signaling system is well known to be particularly important for follicle recruitment or activation in mammals (98). The defects in this signaling pathway are closely associated with female infertility (99, 100). In summary, our transcriptomics data provide strong support to traditional views that ovarian follicle activation or recruitment involves pituitary gonadotropins, steroid hormones and local growth factors.

### Angiogenesis

In addition to signal transduction pathways as described above, one of the most interesting discoveries of the present study was that a large number of biological processes related to angiogenesis or blood vessel formation were enriched in up-regulated genes during PG-PV transition, including angiogenesis, sprouting angiogenesis, vasculogenesis, blood vessel development, blood vessel morphogenesis, lymph vessel formation, response to hypoxia, and regulation of VEGF receptor signaling pathway. The VEGF signaling pathway was also significantly enriched in KEGG pathway analysis as described above. This result suggests that follicle activation or PG-PV transition must involve active angiogenesis or formation of blood vessels, which is likely to ensure sufficient supply of nutrients, hormones and oxygen to support fast growing follicles.

In mammals, the ovary is one of the few organs where physiological angiogenesis occurs in adult, and ovarian angiogenesis has been well documented to be essential for normal follicle growth and maturation (101, 102). A recent study in infantile rats demonstrated a significant increase in VEGF production during preantral-antral transition, further supporting a role for angiogenesis during follicle activation (103). In fish, the oocyte growth involves incorporation of hepatic vitellogenin from the maternal blood stream, making angiogenesis and sufficient blood supply a critical condition for normal folliculogenesis. So far there have been very few studies on ovarian angiogenesis in fish models. In tilapia, both VEGF and its receptor VEGFR2 (Flk-1) were shown to be expressed in the granulosa cells with increasing levels during follicle growth, whereas angiopoietin-2 (Ang-2) and its receptor Tie-2 were expressed in the theca and granulosa cells respectively with expression mostly observed in early SG follicles (104). This agrees well with our transcriptome data in the present study.

### Endocytosis

Different from follicle growth in mammals, which mostly involves proliferation of somatic granulosa and theca cells surrounding the oocyte, the growth of follicles in fish and other oviparous species, especially in the SG stage, is largely due to phenomenal growth of the oocytes because of accumulation of hepatic yolk protein vitellogenin (105). The uptake of vitellogenin from the blood stream by the growing oocytes is mediated by receptor-dependent endocytosis (106, 107). In agreement with this, our pathway analysis revealed endocytosis pathway as one of the major pathways that were significantly enriched with a large number of up-regulated genes. This strongly suggests that although the exogenous vitellogenesis does not occur at PV stage, the increased expression of endocytosis-related genes prepares the follicles for vitellogenin uptake in the subsequent phase of oocyte growth.

### Glycosaminoglycan biosynthesis

The glycosaminoglycans (GAGs) are the most abundant heteropolysaccharides in the body and they are also important components of proteoglycans. The GAG biosynthesis includes two pathways for heparan sulfate/heparin and chondroitin sulfate/dermatan sulfate synthesis respectively. Interestingly, the genes involved in these pathways were significantly increased in PV follicles. It was recently reported that ovarian GAGs promoted gonadotropin-induced follicle development in the mouse, and these GAGs might act, at least partially, by stimulating angiogenesis (108), which agrees with the increased angiogenic activities discussed above. Earlier studies demonstrated that the synthesis of proteoglycans with core proteins and GAGs changed significantly during ovarian folliculogenesis regulated by FSH (109). These studies and our data all suggest important roles for GAGs during folliculogenesis, especially follicle activation.

Although the exact roles of GAGs in PG-PV transition remain unclear, we speculate that they may be related to the biogenesis of the cortical alveoli (CA) or cortical vesicles/granules in the oocytes, which is a major hallmark of PV stage as compared to PG (66, 110). The cortical alveoli play an important role in cortical reaction during fertilization (111), and their contents contain rich glycosylated materials (112) including glycoproteins such as polysialoglycoprotein (113, 114) and GAG-like substances (115).

### RNA silencing

Our GO and KEGG analyses showed that most enriched biological processes and pathways contained up-regulated genes during PG-PV transition, suggesting increased biological activities in the process. However, some GO terms were significantly enriched for down-regulated genes, including *piRNA metabolic process* and *gene silencing by RNA*. piRNAs (Piwi-interacting RNAs) are a special group of small RNAs (24–31 nt long) that are associated with the Piwi proteins, a subgroup of the Argonaute family. These small RNAs and Piwi are specifically expressed in the germ cells to target transposons and other genes (116). Evidence has accumulated that piRNAs play important roles in germ cell development, embryogenesis and genome stability (117) although the importance has not been supported by Piwi knockout study in the mouse (118).

In addition to piRNA, microRNA-mediated RNA silencing has been reported to play important roles in ovarian folliculogenesis (119). In a recent transcriptome study, we characterized the profiles of microRNAs during the PG-PV transition in the zebrafish ovary. We identified a 13-microRNA expression signature that showed significant changes during the transition (120). Although the role of non-coding small RNAs in zebrafish folliculogenesis remains largely unknown, the enrichment of both piRNA metabolic process and gene silencing by RNA in down-regulated genes during the PG-PV transition strongly suggests an important role for RNA interference or silencing in follicle activation. This would be an interesting direction for research in the future.

### Proteolysis

Although it was not as significant as other GO terms, the proteolysis process was enriched by a large number of down-regulated genes during PG-PV transition. This is interesting and has promoted us to hypothesize that an active protein turnover may occur in PG and/or early PV follicles; however, the activity of protein degradation decreases when the follicles approach the end of PV stage (our sampling stage) prior to entering vitellogenic growth. Our unpublished proteomics analysis also showed a dramatic change in protein profile during PG-PV transition in contrast to the relatively consistent and stable profiles afterwards in vitellogenic growth (Lau and Ge, unpublished data).

### Oocytes vs. follicle cells

In addition to biological process and pathway enrichment analyses during PG-PV transition, we also looked at the expression changes of genes that are expressed in the two different compartments of ovarian follicles, i.e., oocytes and follicle cells. Since the information regarding expression localization within the follicles is limited in the zebrafish, our analysis was mostly based on previously published data in the zebrafish and a dataset available in the sheep with the assumption that most genes also have similar spatial distribution in the zebrafish follicle (65). Interestingly, as we observed in our previous studies, most oocyte-expressed genes were stable in their expression during follicle activation except a few; in contrast, a large number of genes in the follicle cells showed dramatic changes in expression, mostly up-regulated, during PG-PV transition. This suggests that the regulation of oocyte-expressed genes may likely occur at post-transcriptional level whereas those in the follicle cells are mostly controlled at the transcription level. The observation that many up-regulated genes that showed the most significant changes in the follicle cells were related to signaling of endocrine and paracrine factors strongly suggests that the somatic follicle cells are the site where various signaling molecules act and interact to control folliculogenesis.

In summary, the transcriptome analysis in the present study for differentially expressed genes during follicle activation provides critical insights into the candidate molecules involved in early folliculogenesis, and in particular, the regulatory networks that play roles in triggering and driving follicle activation and puberty onset. As expected, a large number of signal transduction pathways were activated during PG-PV transition or follicle activation, which may be responsible for signaling of endocrine hormones and paracrine factors such as gonadotropins, steroids, and growth factors. In addition, a variety of pathways concerning angiogenesis or blood vessel formation were also activated during follicle activation, indicating increased demand for supplying nutrients, hormones, and oxygen to the growing follicles. Other pathways responsible for specific events or processes such as biogenesis of cortical alveoli and vitellogenin uptake were also significantly enriched. The follicle activation or PG-PV transition may also involve other regulatory mechanisms such as RNA silencing and proteolysis. With the powerful genome editing technologies (TALEN and CRISPR/Cas9) available now in the zebrafish model, the functions of novel genes identified can be further validated by targeted loss-of-function approach.

## Ethics statement

This study was carried out in accordance with the recommendations by the Legislative Council of Macao Special Administrative Region under Article 71 (1) of the Basic Law. The protocol was approved by the Research Ethics Committee of the University of Macau.

## Author contributions

BZ designed and executed all the experiments, writing the manuscript. LP: data analysis. YC: data analysis. WG: experimental design, writing the manuscript.

### Conflict of interest statement

The authors declare that the research was conducted in the absence of any commercial or financial relationships that could be construed as a potential conflict of interest.
