# Supplementary material for: Transcriptomic Analysis for Differentially Expressed Genes in Ovarian Follicle Activation in the Zebrafish
Source: Front Endocrinol (Lausanne). 2018 Oct 11;9:593. doi: 10.3389/fendo.2018.00593 (PMC6193065; doi:10.3389/fendo.2018.00593)
Supplement: Supplementary file 1 [file Table_1.DOCX]

Table 1. Primers used in real-time qPCR

| Gene | Accession no. | Primer no. | Sequence | Expected size |
| --- | --- | --- | --- | --- |
| *ef1a* | NM_131263 | 728 | GGCTGACTGTGCTGTGCTGATTG | 409 |
|  |  | 729 | CTTGTCGGTGGGACGGCTAGG |  |
| *cyp19a1a* | NM_131154 | 818 | TGTGCGTGTCTGGATCAATGG | 220 |
|  |  | 819 | AAGCCCTGGACCTGTGAGAG |  |
| *fshr* | NM_001001812 | 957 | AACATGCACATAGAGAGGATTCCCAG | 334 |
|  |  | 958 | GCTCAGTAAACAGCTCCAGGC |  |
| *inha* | XM693951 | 520 | AGCCTCCTCTGCCAGTGTTG | 301 |
|  |  | 521 | ATGTTGATGGAAGCGATGGTCTC |  |
| *inhbaa* | ENSDARG00000012671 | 931 | AACAGGCAGAACAGACGGAGATC | 180 |
|  |  | 932 | GCAGCCGAATGTTGACGTTAGC |  |
| *notch3* | NM_131544 | 1188 | TAAGGATGAGATAGATGAGTGCCAGTC | 199 |
|  |  | 1189 | ACAGTCACACCGATAGCCATTAGG |  |
| *amh* | AY677080 | 641 | ATGAAGTGTTGATGAGAAAGGTGGATG | 323 |
|  |  | 642 | TACAGTCGGCGTGGAGGAATTG |  |
| *gadd45ga* | NM_205691 | 4253 | CCGGAACAGCACTAGAGGAG | 127 |
|  |  | 4254 | GTAGCCAGAACGCAGAAAGC |  |
| *lpl* | NM_1311277 | 4263 | CCTCGGGATTGGAAACTACA | 153 |
|  |  | 4264 | AGCACAGGTCGTCTTCAGGT |  |
| *MARCH4* | NM_001030165 | 4259 | GTTCTCCAGAGACCGCAAAG | 116 |
|  |  | 4260 | GACACGGACTCAACAGCTCA |  |
| *ube2ql1* | XM_680552 | 4261 | CTTCTCTCCTCCGTTCATGC | 170 |
|  |  | 4262 | GACCCTGTCCTTTCACCAGA |  |
| *zp2.1* | NM_001039883 | 4255 | CCTGGTTTGGTGCTTTTTGT | 156 |
|  |  | 4256 | GAAGTGGTTGGCTGACCTGT |  |
| *zp2.5* | NM_131330 | 4257 | CCTGGTTTGGTGCTTTTTGT | 147 |
|  |  | 4258 | GAAGTGGTTGGCTGACCTGT |  |
