# Supplementary material for: Transcriptomic Analysis for Differentially Expressed Genes in Ovarian Follicle Activation in the Zebrafish
Source: Front Endocrinol (Lausanne). 2018 Oct 11;9:593. doi: 10.3389/fendo.2018.00593 (PMC6193065; doi:10.3389/fendo.2018.00593)

Up-regulated Pathways

Glycosaminoglycan biosynthesis – chondroitin sulfate/dermatan sulfate

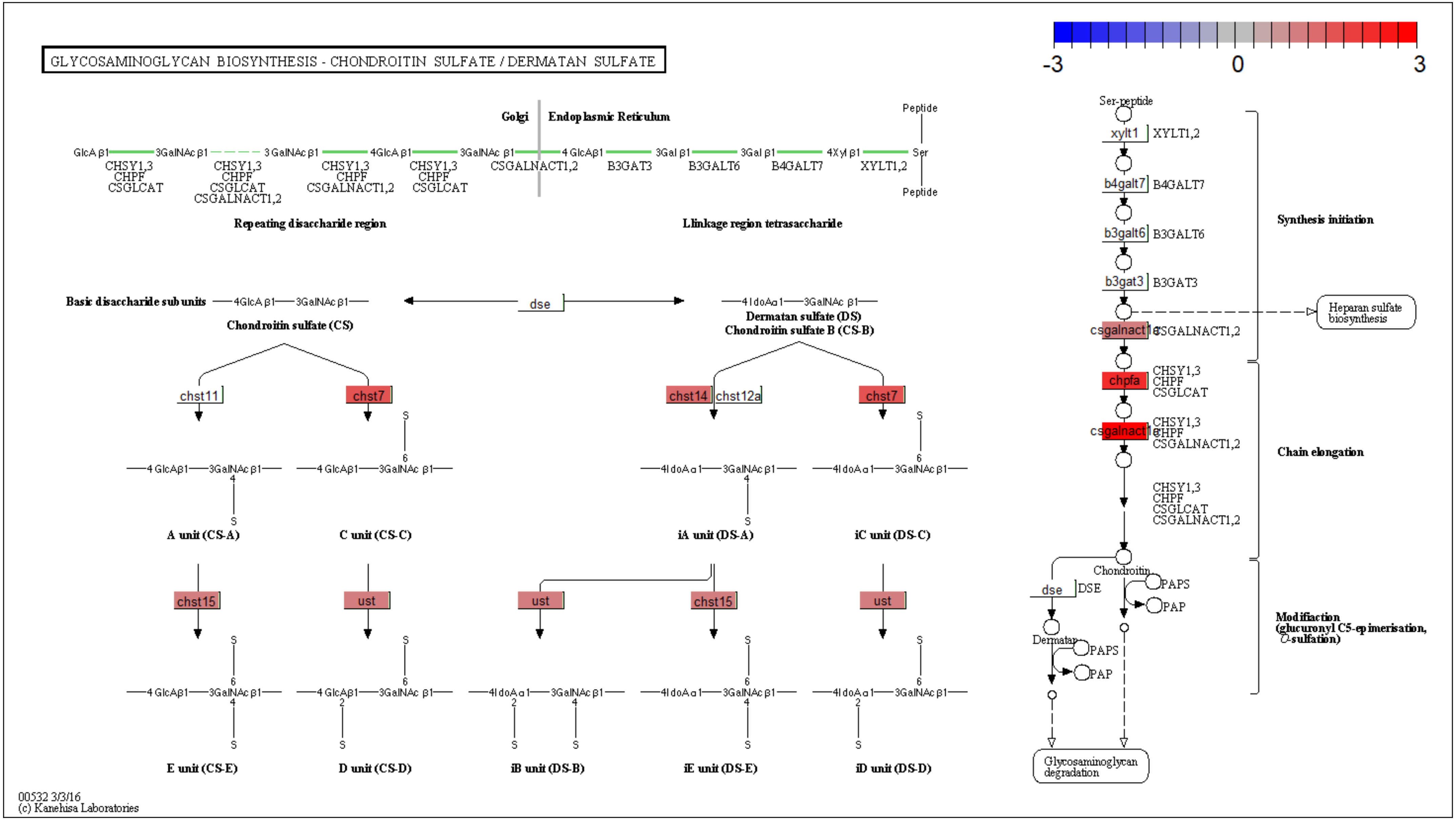

Steroid biosynthesis

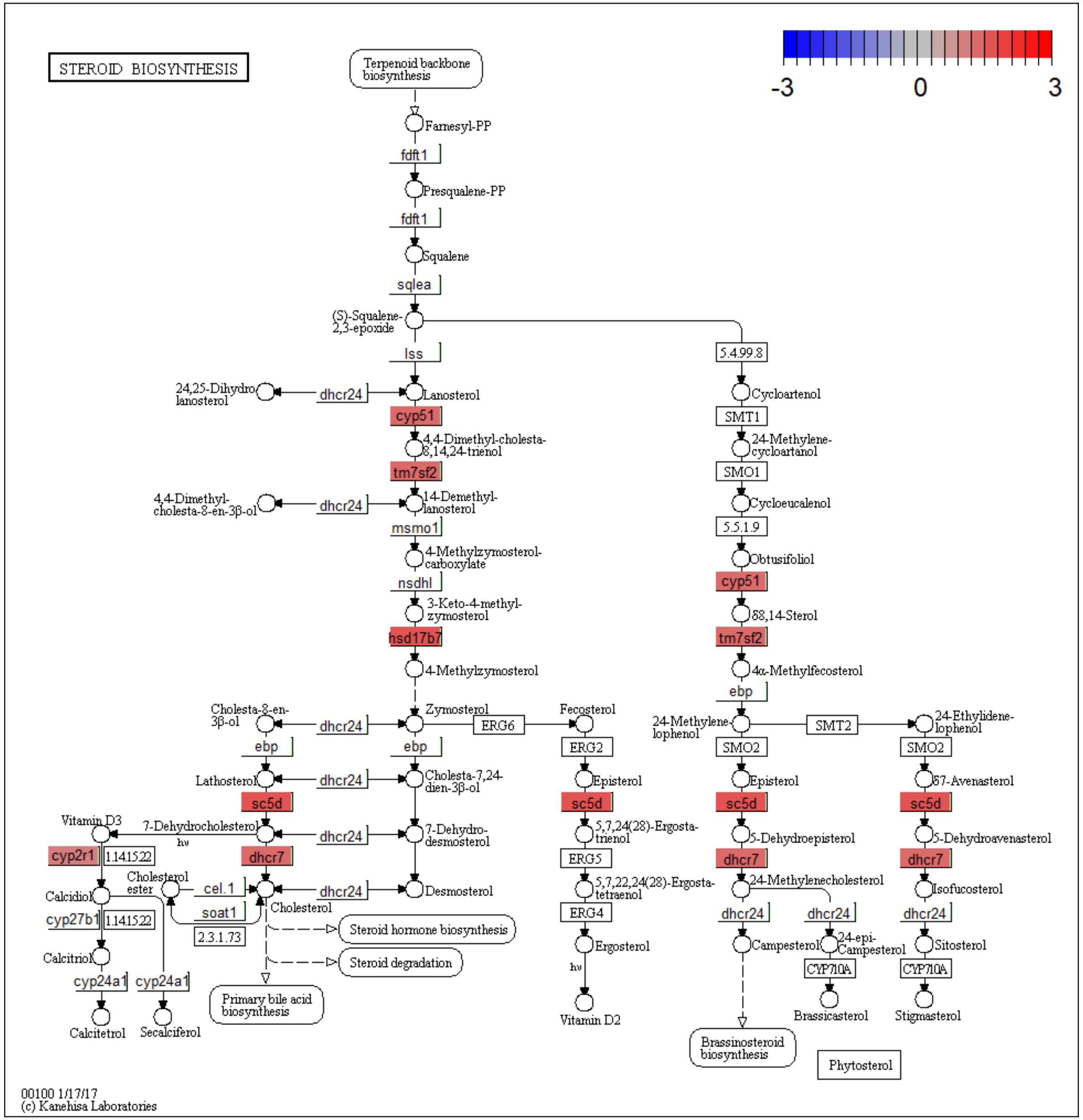

Inositol phosphate metabolism

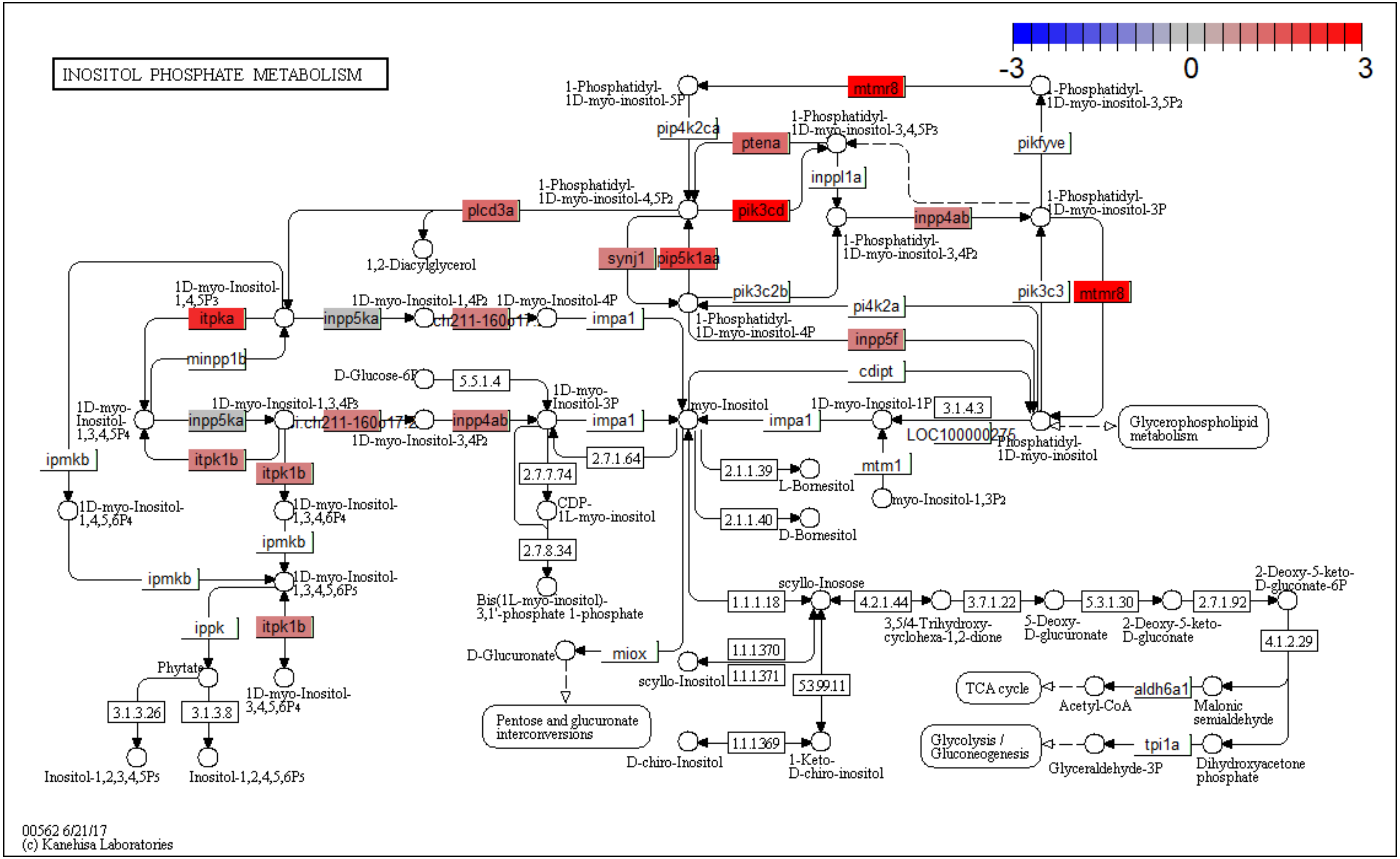

Phosphatidylinositol signaling system

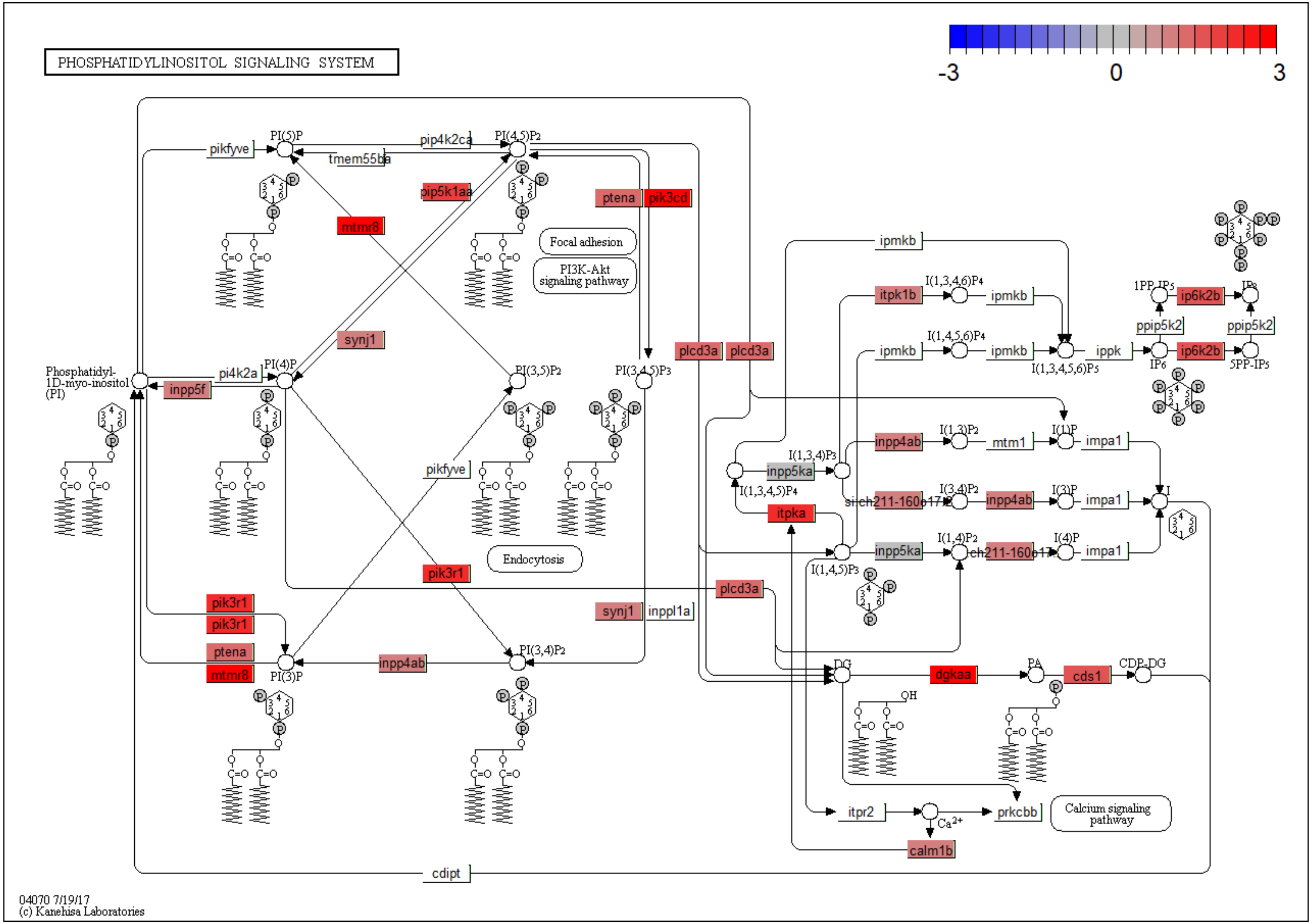

MAPK signaling pathway

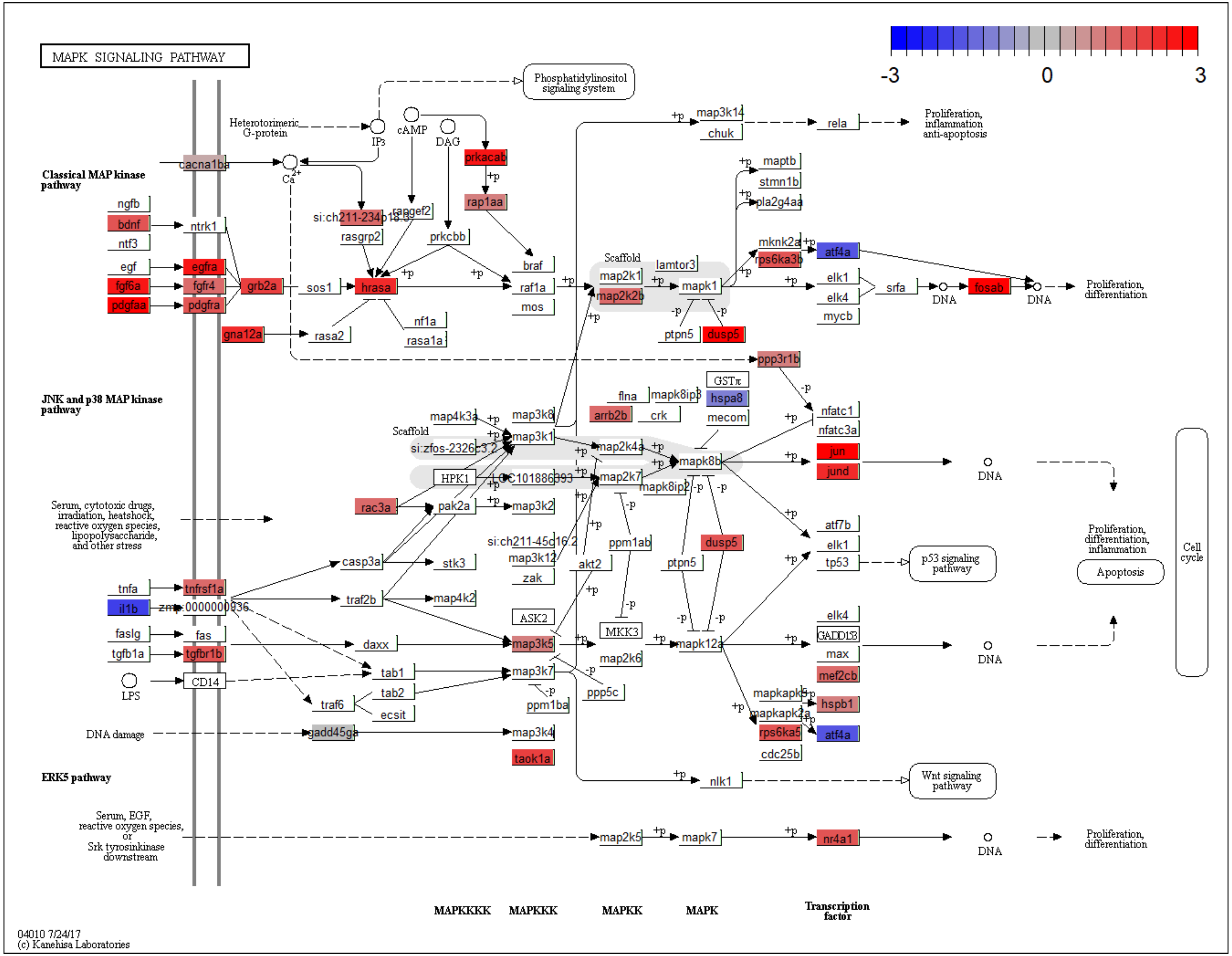

ERBB signaling pathway

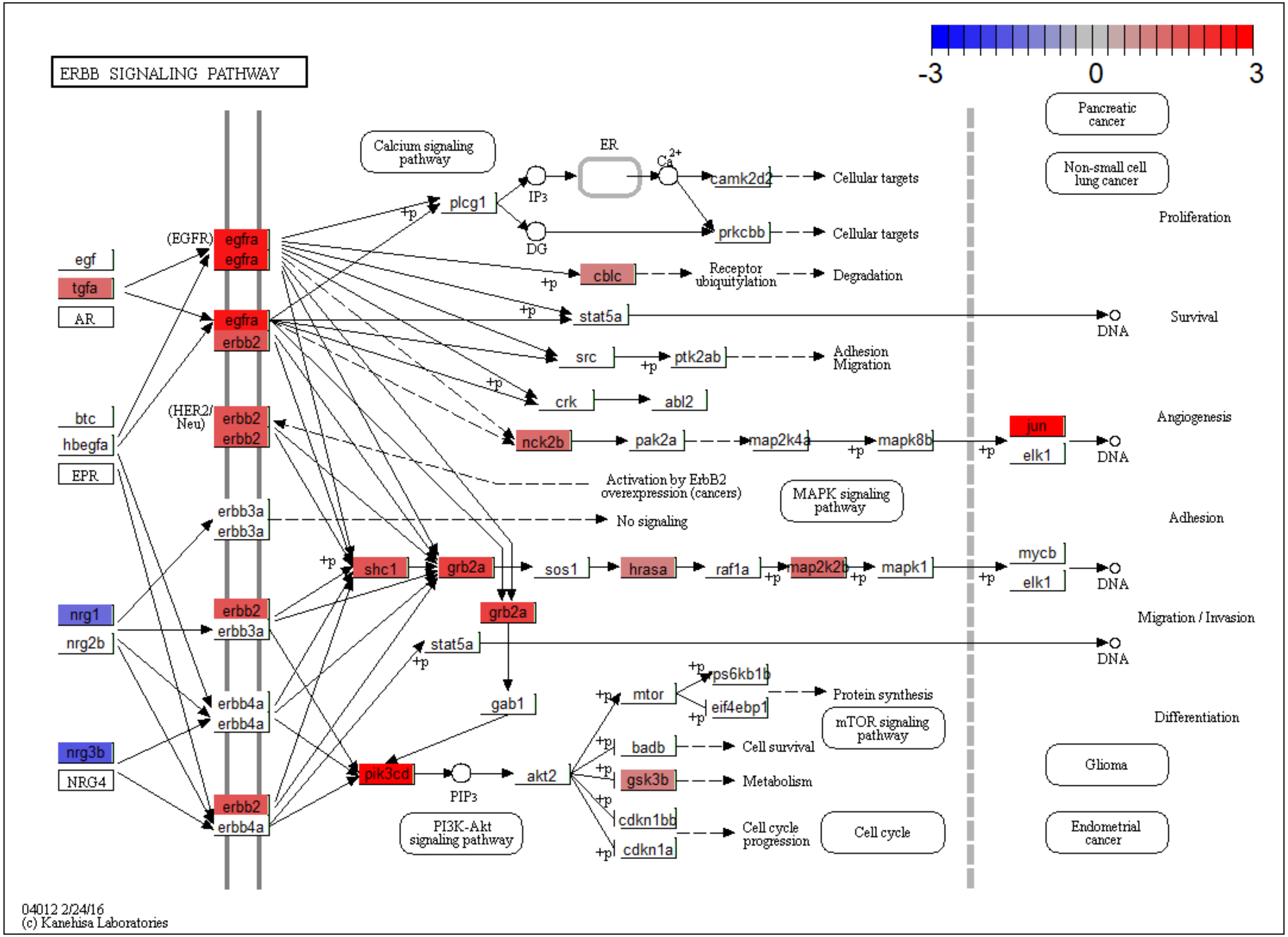

TGF-beta signaling pathway

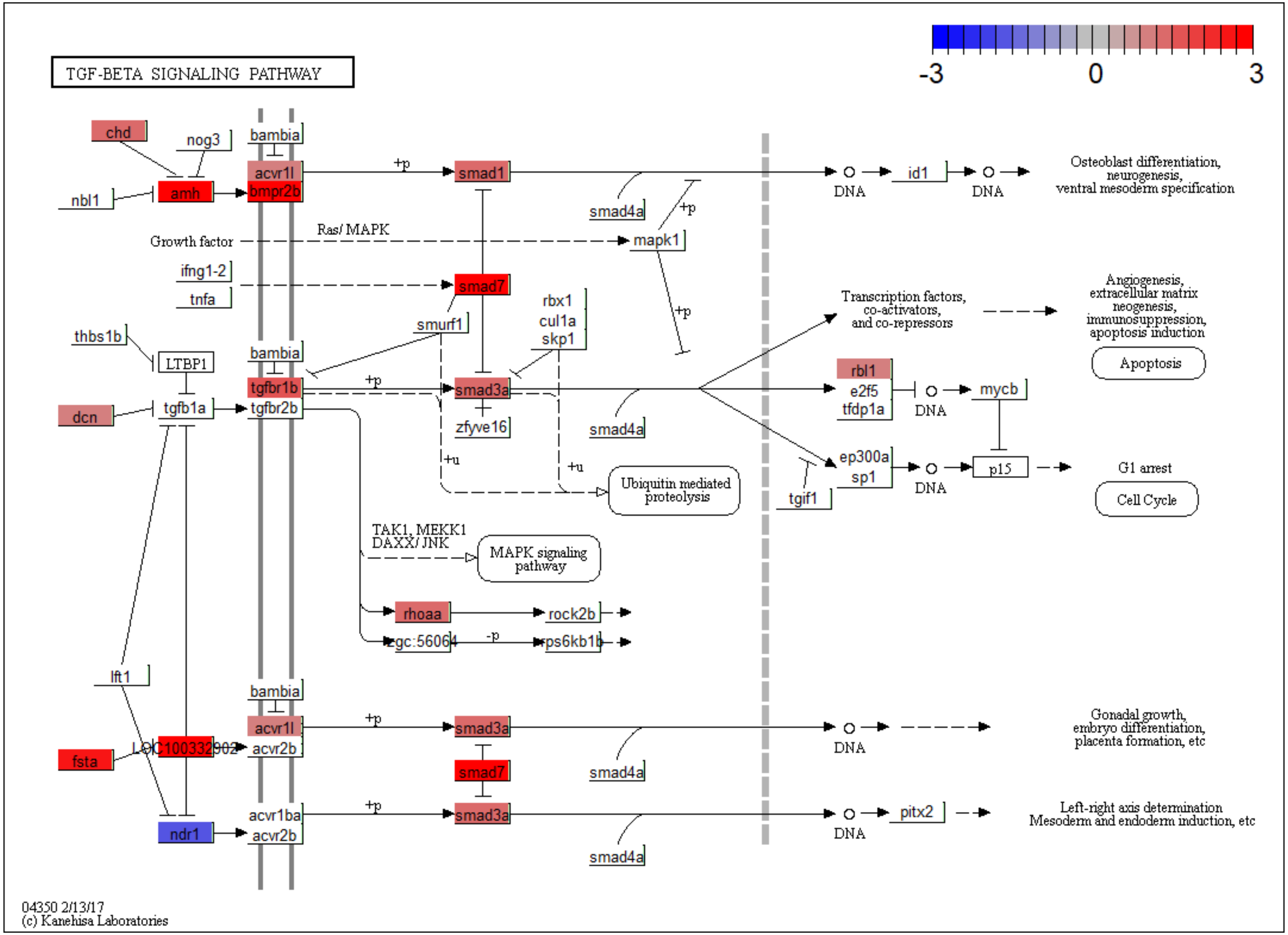

VEGF signaling pathway

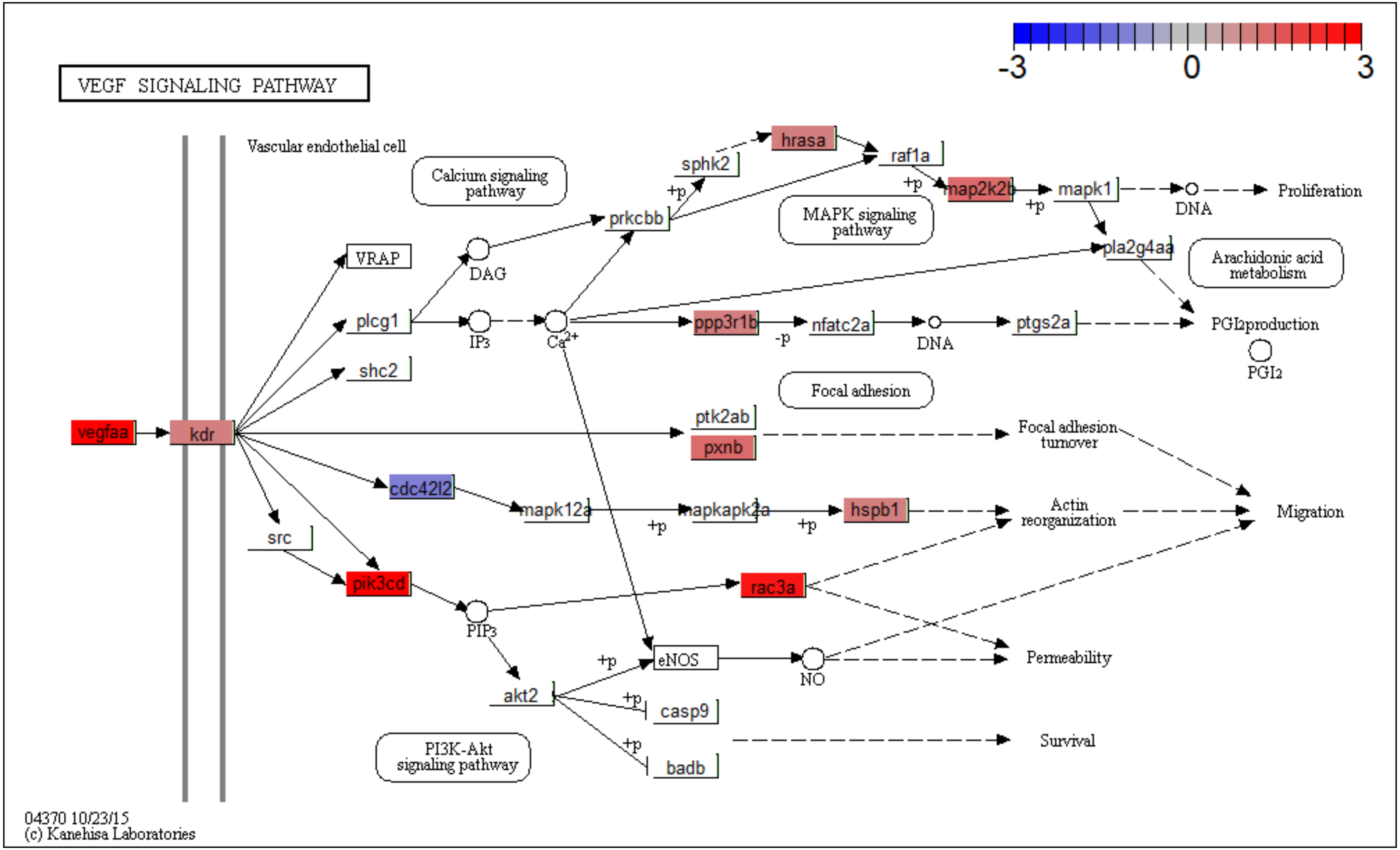

Foxo signaling pathway

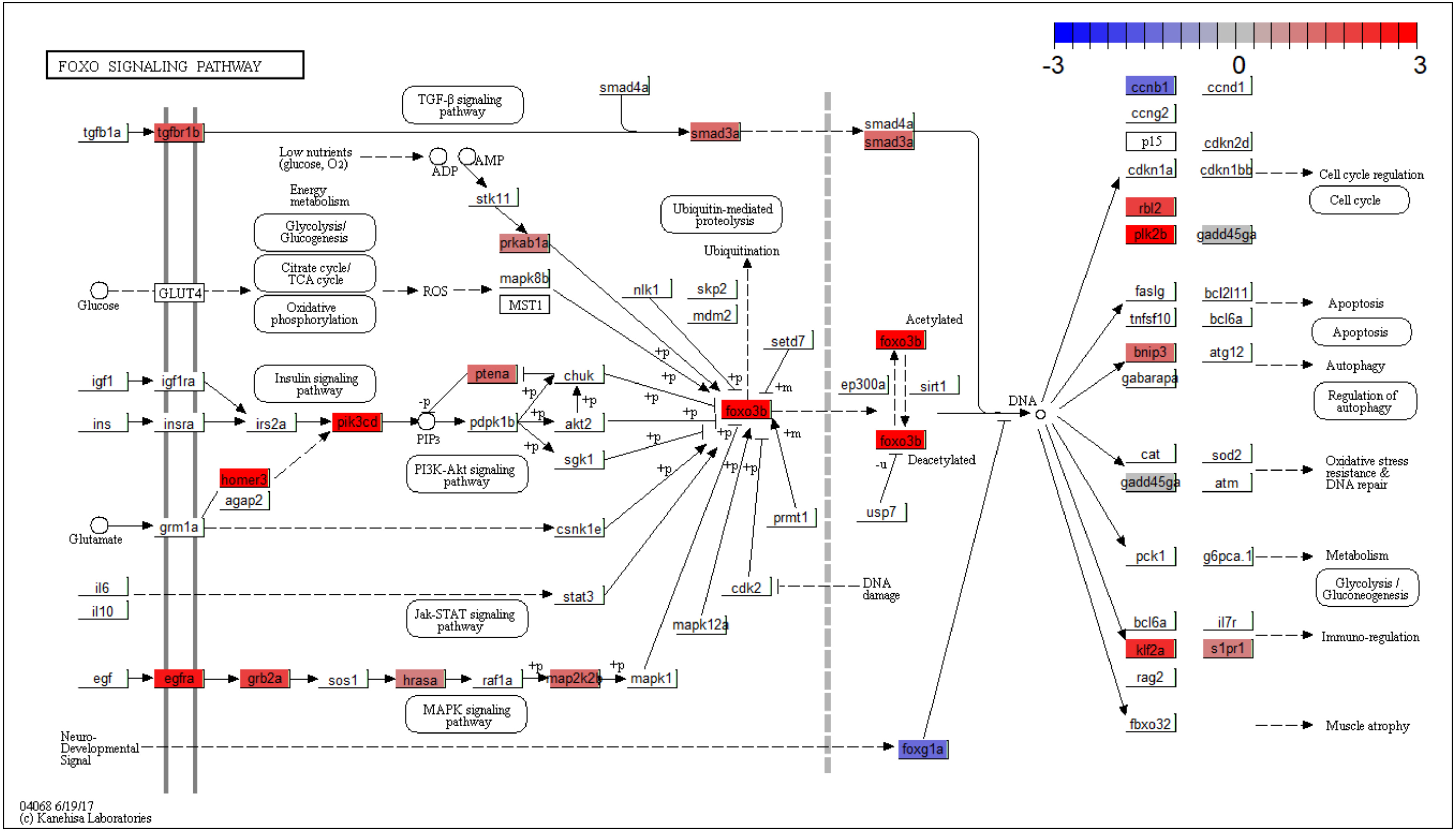

Hedgehog signaling pathway

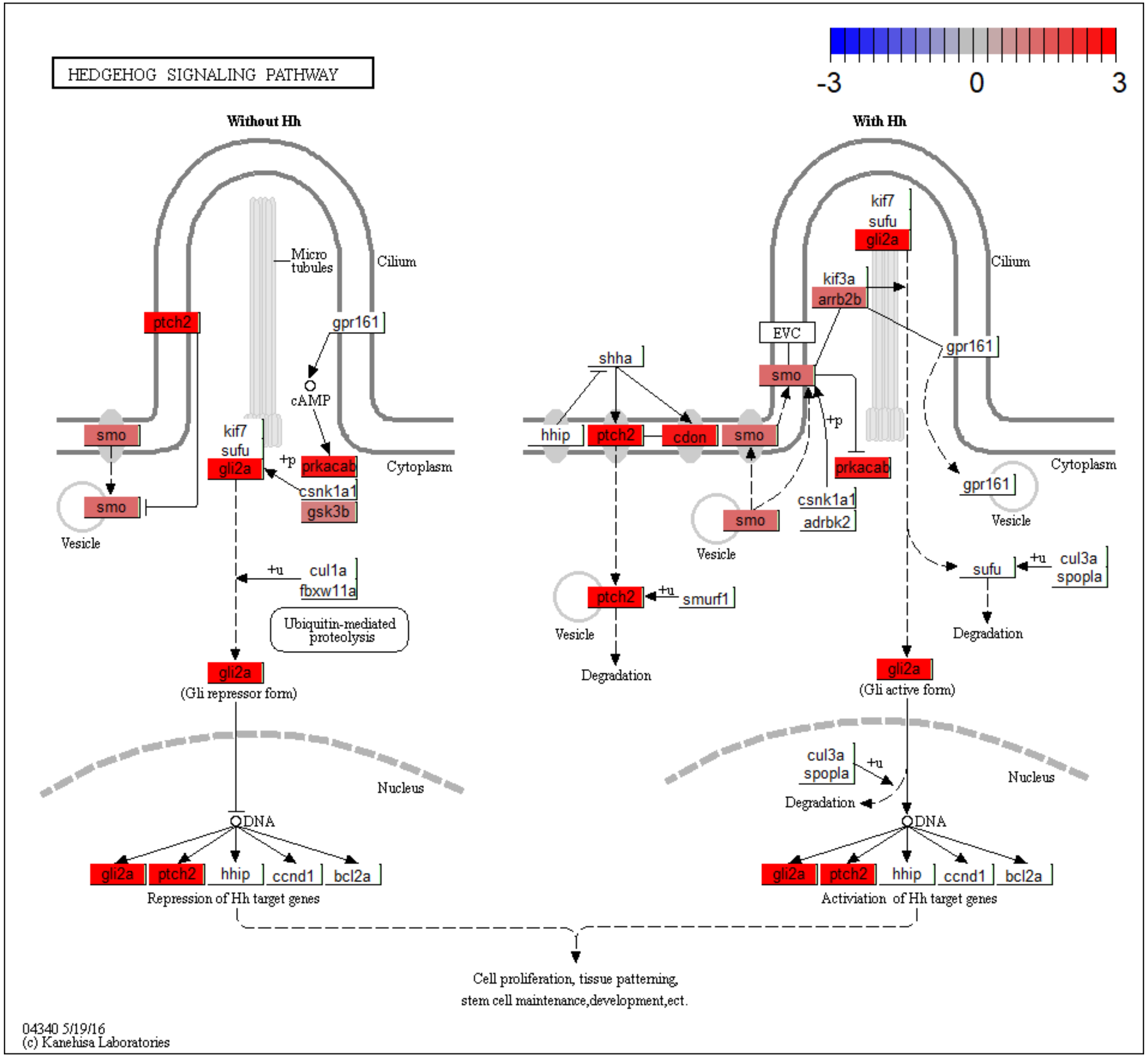

Cytokine-cytokine receptor interaction

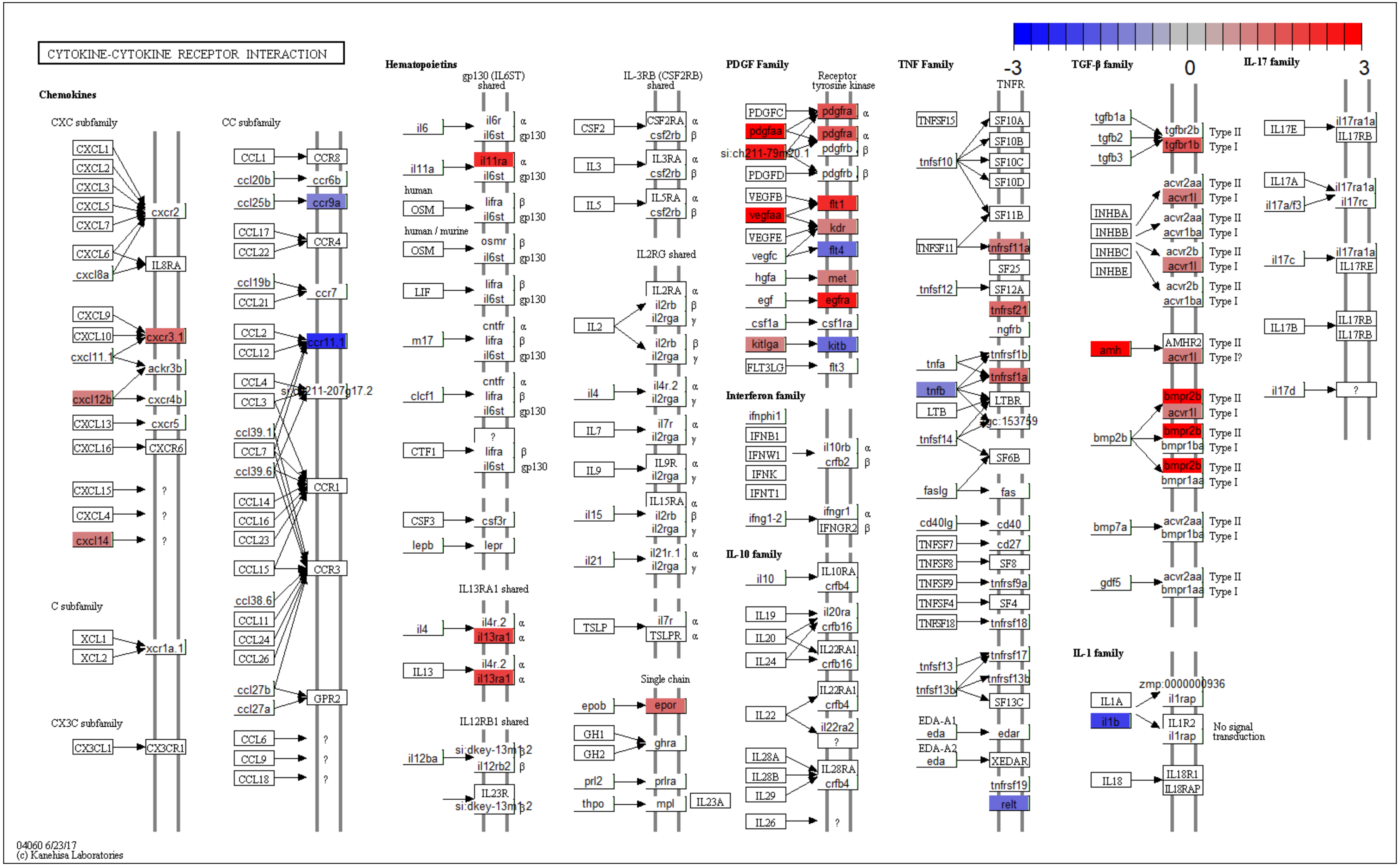

ECM-receptor interaction

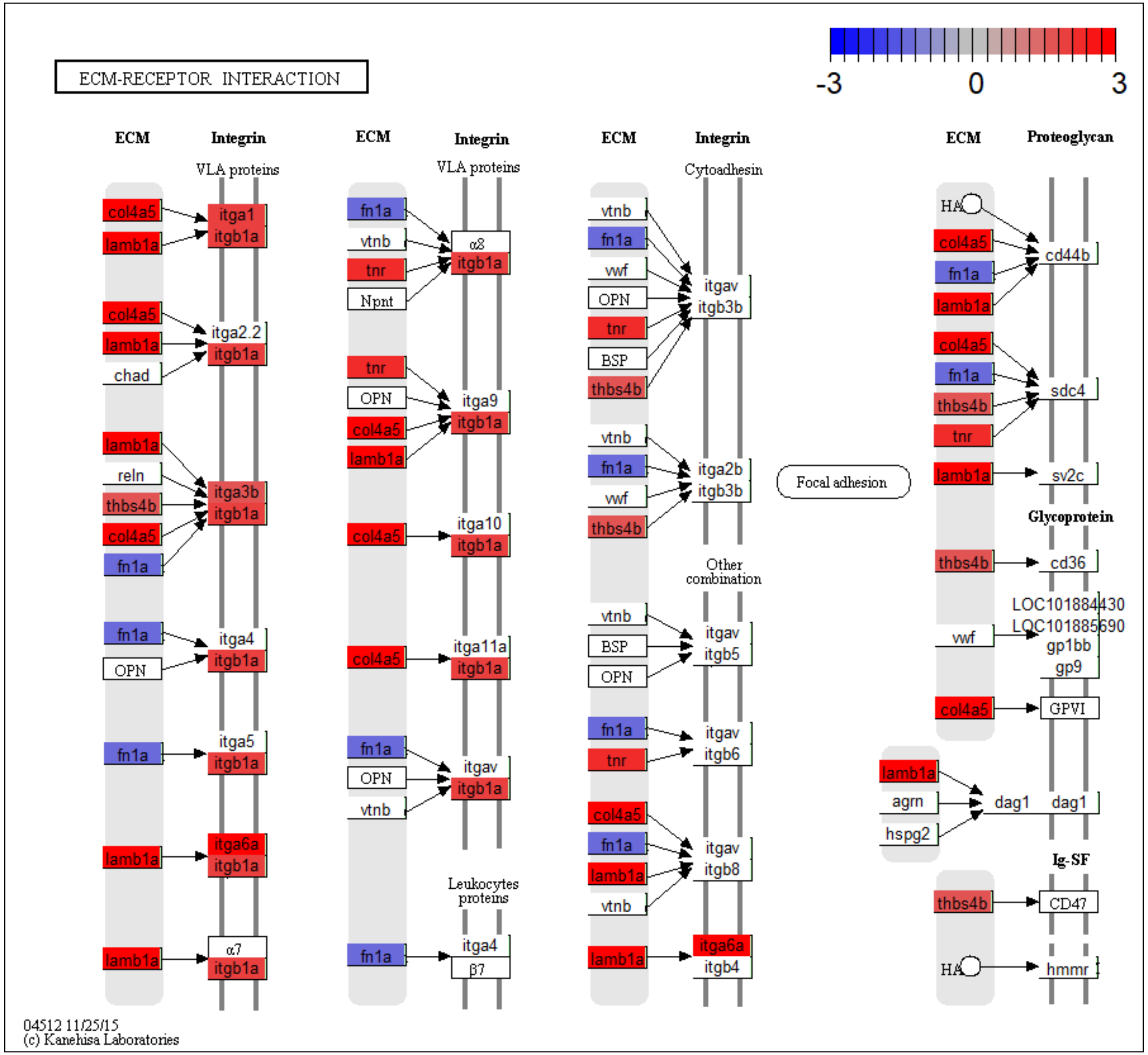

Endocytosis

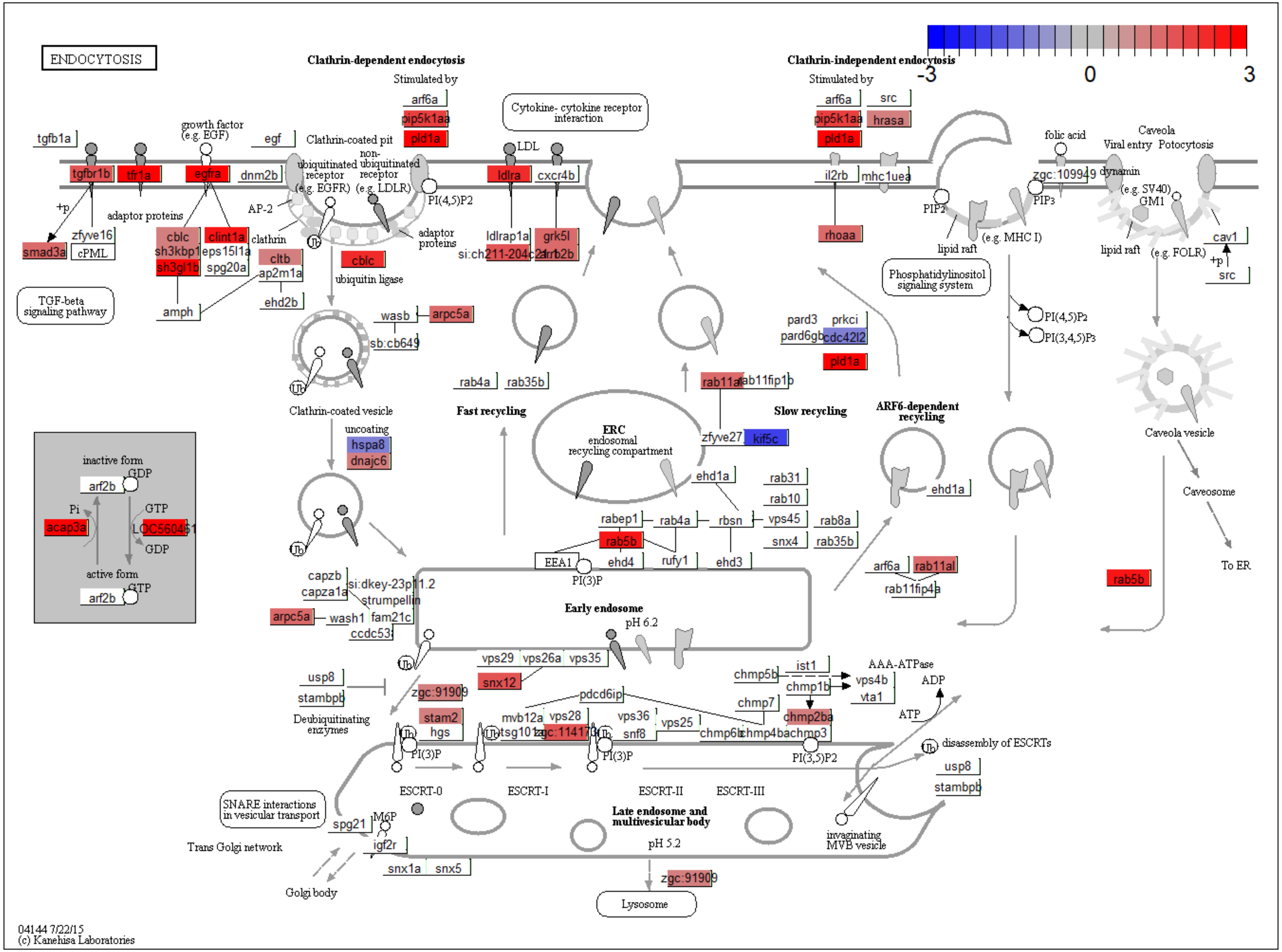

Pentose phosphate pathway

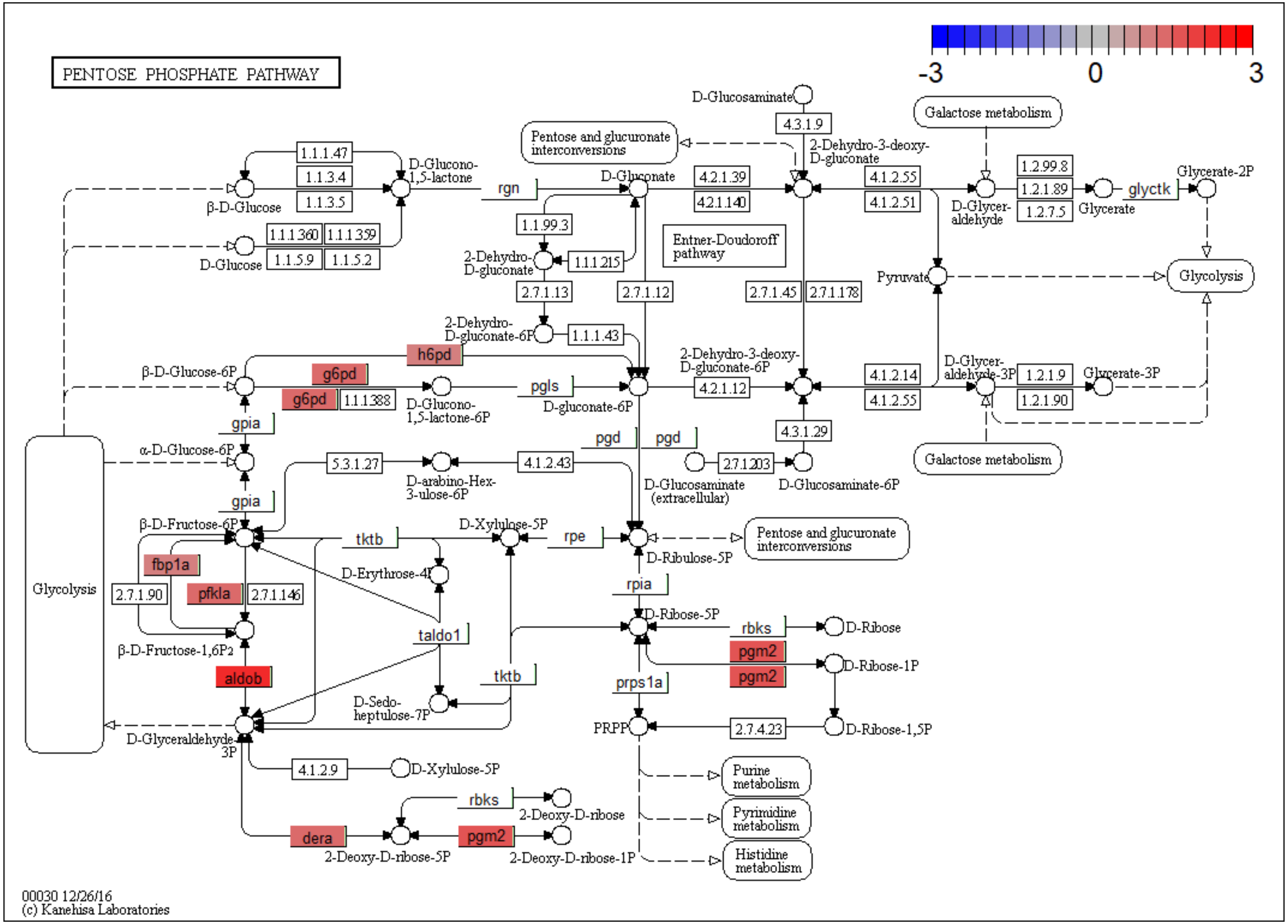

Glycerophospholipid metabolism

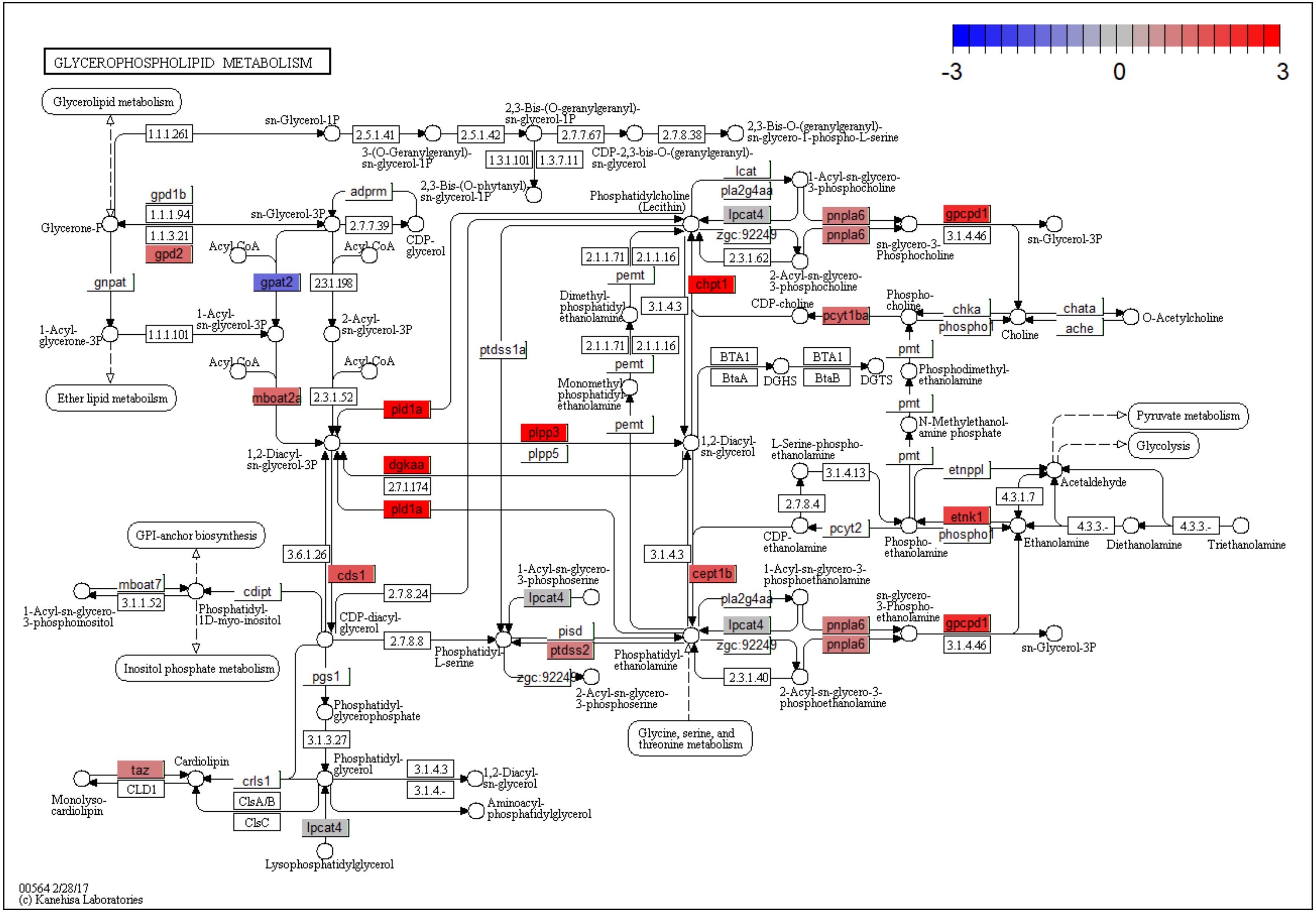

Focal adhesion

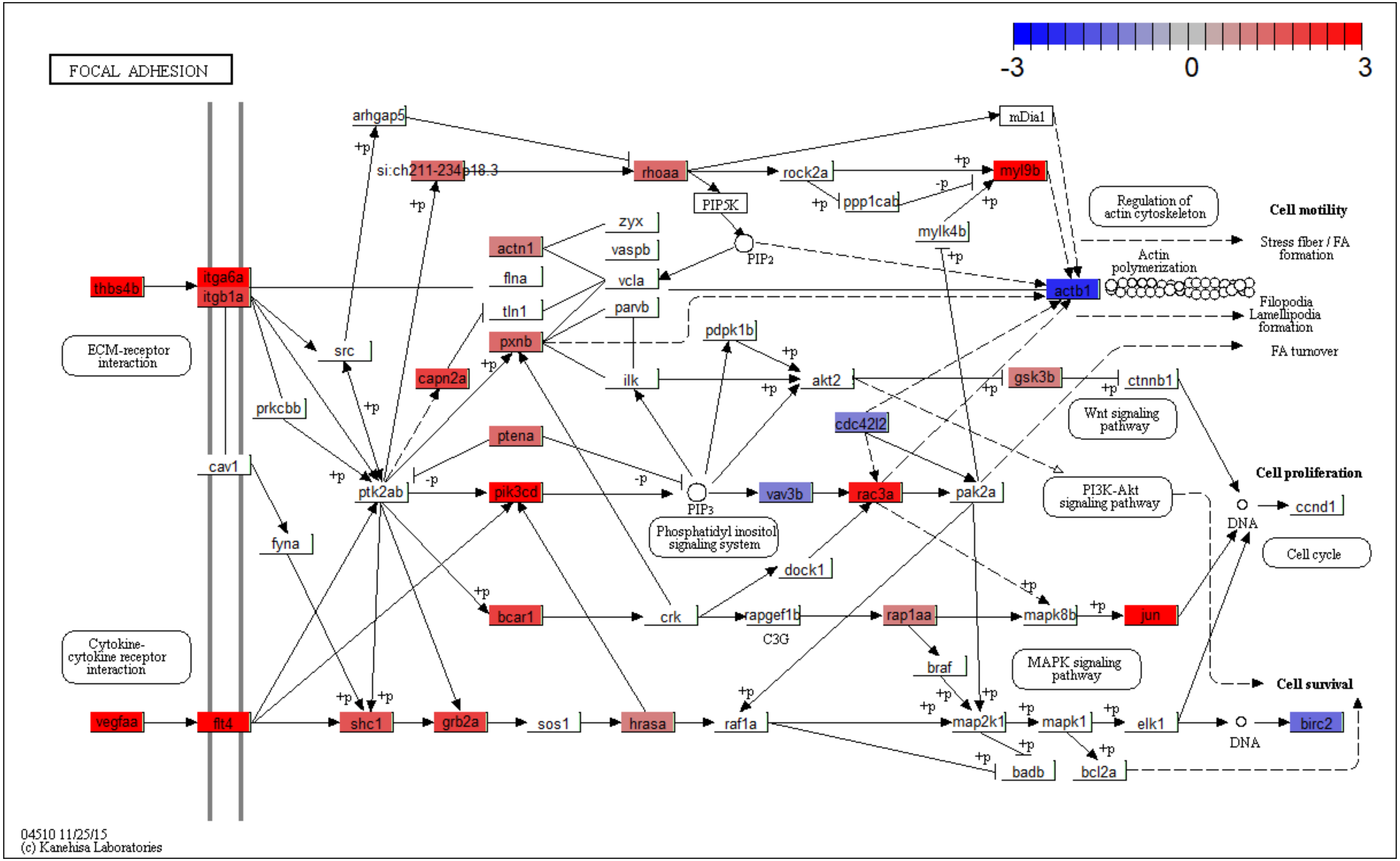

Regulation of actin cytoskeleton

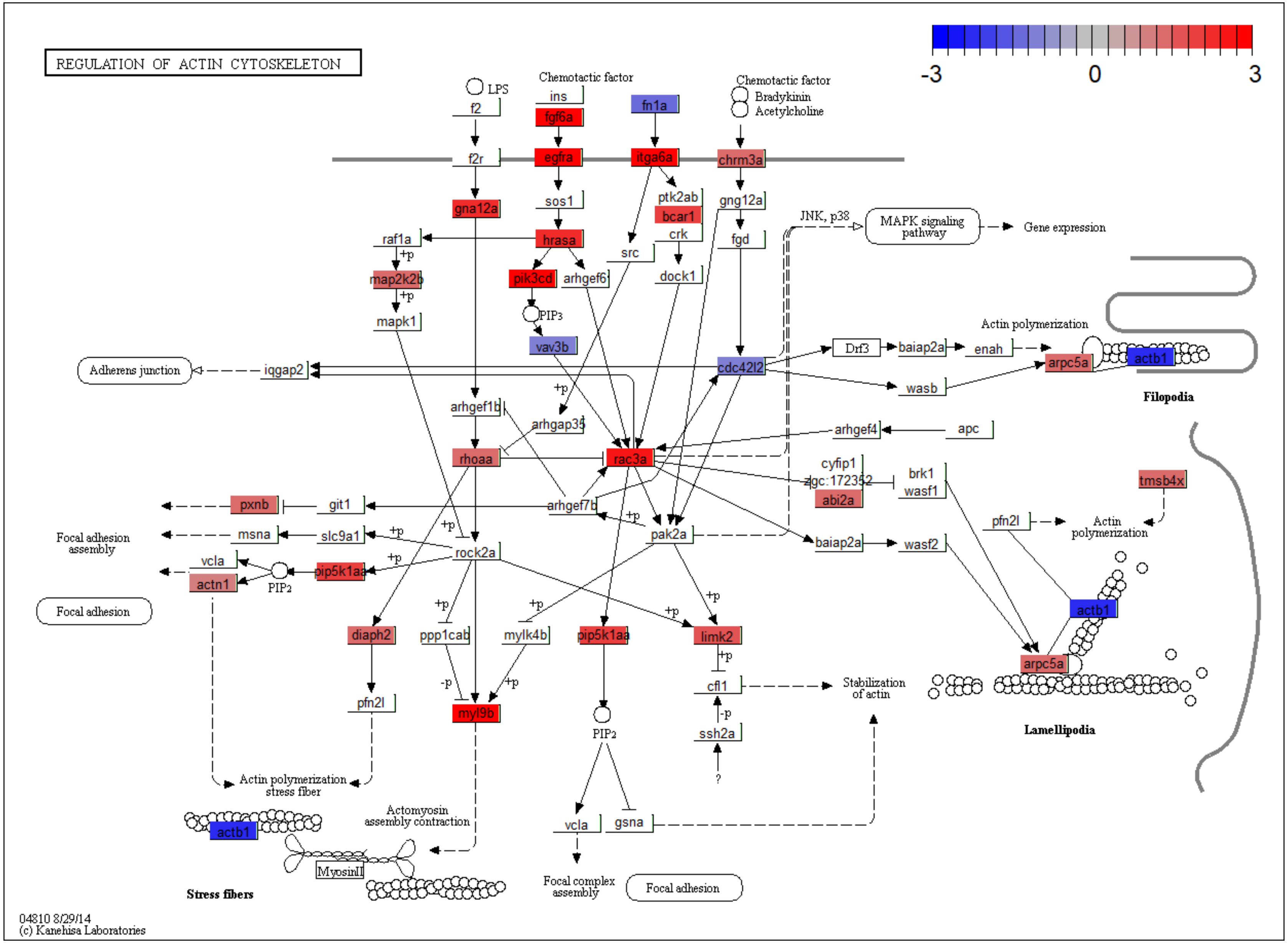

Supplement: Supplementary file 3 [file Data_Sheet_1.pdf]
