# Supplementary figures and images for: Transcriptomic Analysis for Differentially Expressed Genes in Ovarian Follicle Activation in the Zebrafish
Source: Front Endocrinol (Lausanne). 2018 Oct 11;9:593. doi: 10.3389/fendo.2018.00593 (PMC6193065; doi:10.3389/fendo.2018.00593)

# Down-regulated Pathways

## Tryptophan metabolism

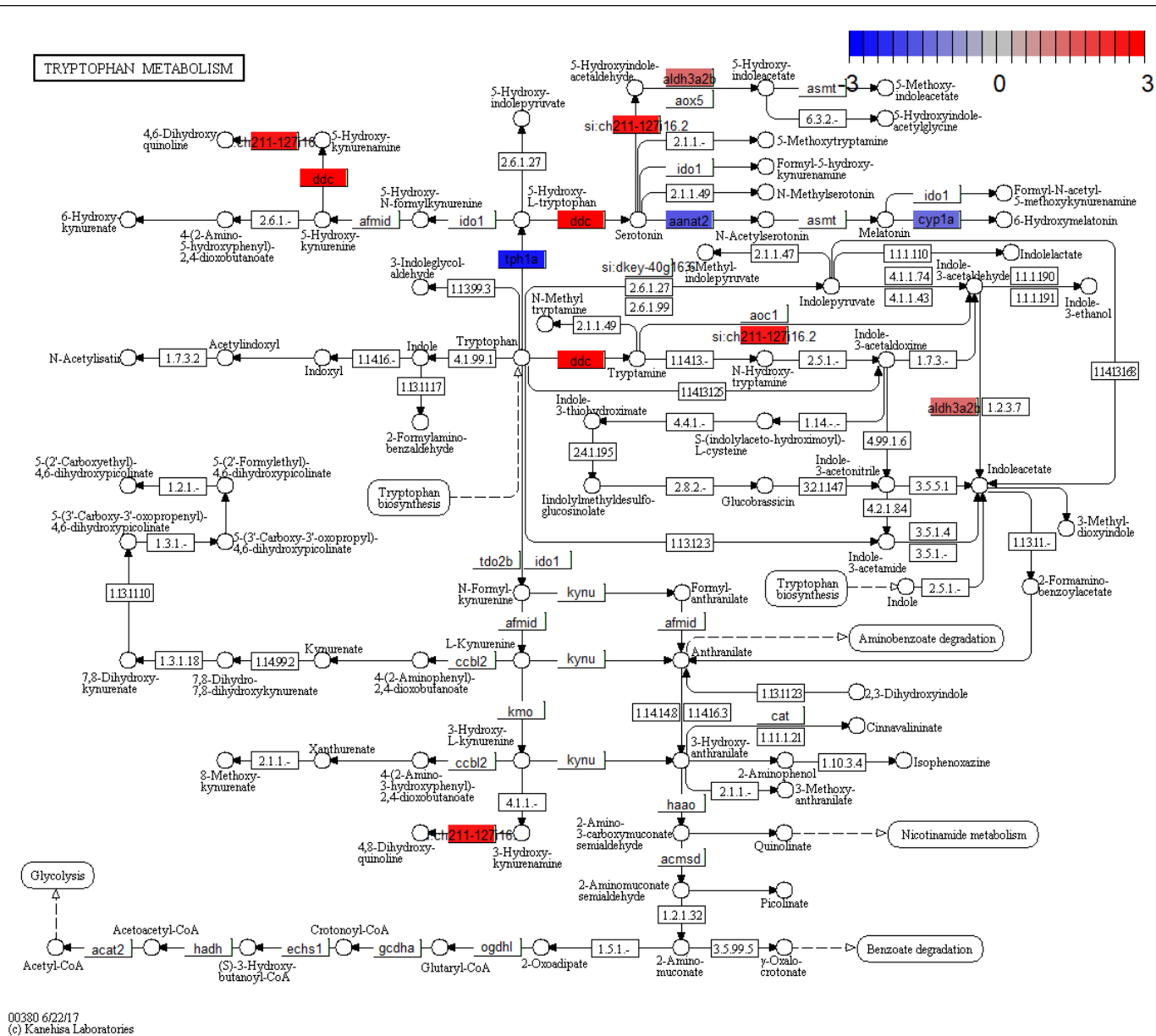

# Tight junction

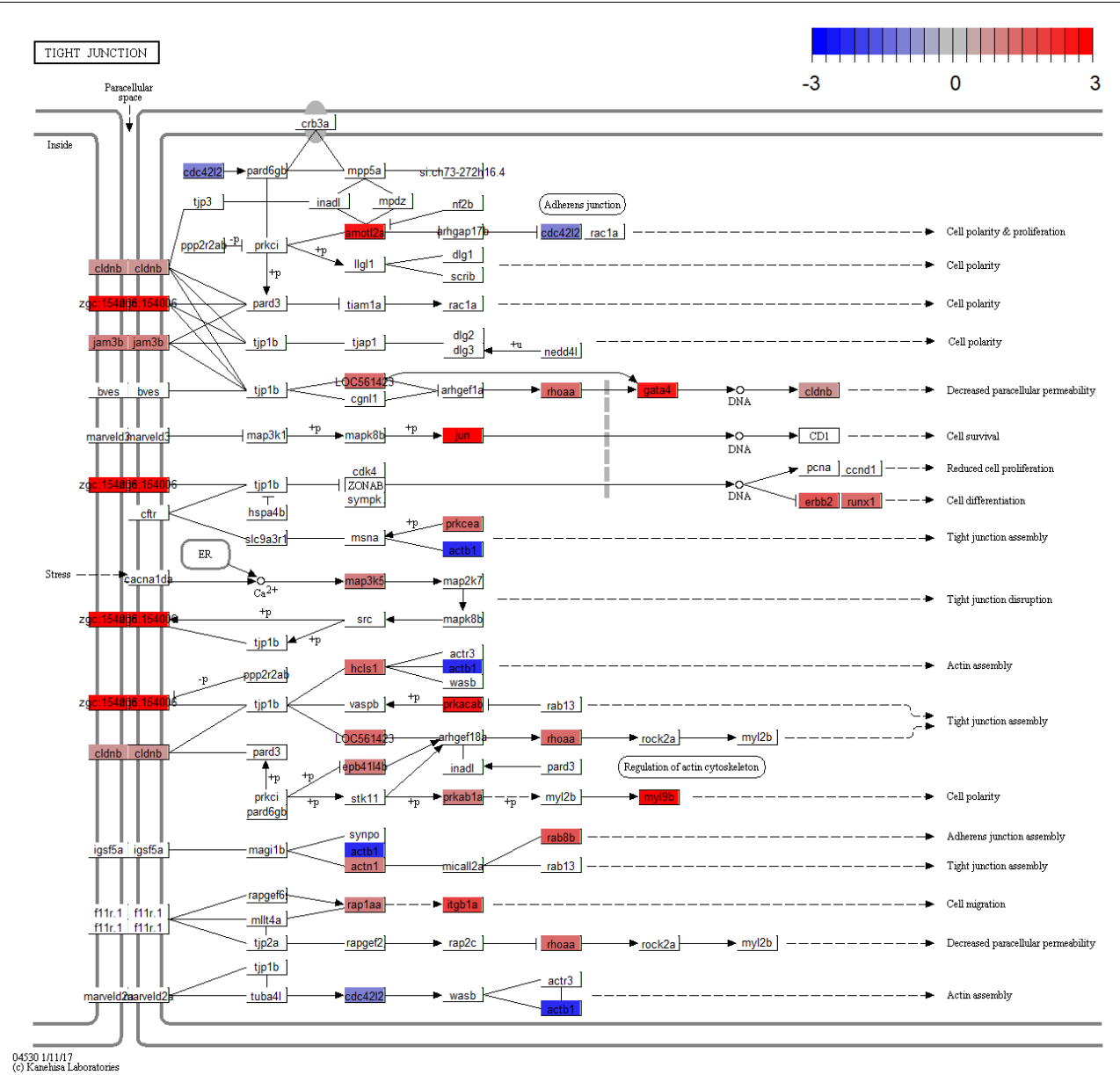

Supplement: Supplementary file 4 [file Data_Sheet_2.pdf]
